# Biosynthesis of prostaglandin 15dPGJ_2_ -glutathione and 15dPGJ_2_-cysteine conjugates in macrophages and mast cells via MGST3

**DOI:** 10.1016/j.jlr.2022.100310

**Published:** 2022-11-09

**Authors:** Julia Steinmetz-Späh, Jianyang Liu, Rajkumar Singh, Maria Ekoff, Sanjaykumar Boddul, Xiao Tang, Filip Bergqvist, Helena Idborg, Pascal Heitel, Elin Rönnberg, Daniel Merk, Fredrik Wermeling, Jesper Z. Haeggström, Gunnar Nilsson, Dieter Steinhilber, Karin Larsson, Marina Korotkova, Per-Johan Jakobsson

**Affiliations:** 1 Division of Rheumatology, Department of Medicine, Karolinska Institutet and Karolinska University Hospital, Stockholm, Sweden; 2 Division of Physiological Chemistry 2, Department of Medical Biochemistry and Biophysics, Karolinska Institutet, Stockholm, Sweden; 3 Division of Immunology and Allergy, Department of Medicine, Karolinska Institutet and Karolinska University Hospital, Stockholm, Sweden; 4 Institute of Pharmaceutical Chemistry, Goethe-University Frankfurt, Frankfurt, Germany

**Keywords:** PGD_2_/15-deoxy-Δ^12,14^-prostaglandin J_2_, cyclopentenones, lipid metabolism, GSH, resolution, inflammation, mPGES-1 inhibition, microsomal glutathione S-transferase-3, Michael adducts, immune cells, 15dPGJ_2_, 15-deoxy-Δ^12,14^-prostaglandin J_2_, 15dPGJ_2_-Cys, 15dPGJ_2_-cysteine, 15dPGJ_2_-GS, 15dPGJ_2_-glutathione, BMDM, bone marrow derived macrophages, CBMCs, cord-blood derived mast cells, COX, cyclooxygenase, DDM, dodecyl β-D-maltoside, FBS, fetal bovine serum, FLAP, 5-lipoxygenase activating protein, GST, glutathione S-transferase, LPS, lipopolysaccharides, LTC4S, leukotriene C4 synthase, MAPEG, membrane associated proteins involved in eicosanoid and glutathione metabolism, M-CSF, Macrophage Colony-Stimulating Factor, MGST, microsomal glutathione S-tranferase, MQ water, MilliQ water, mPGES-1, microsomal prostaglandin E synthase-1, MRM, multiple reaction monitoring, PGD_2_, prostaglandin D_2_, PGE_2_, prostaglandin E_2_

## Abstract

Inhibition of microsomal prostaglandin E synthase-1 (mPGES-1) results in decreased production of proinflammatory PGE_2_ and can lead to shunting of PGH_2_ into the prostaglandin D_2_ (PGD_2_)/15-deoxy-Δ^12,14^-prostaglandin J_2_ (15dPGJ_2_) pathway. 15dPGJ_2_ forms Michael adducts with thiol-containing biomolecules such as GSH or cysteine residues on target proteins and is thought to promote resolution of inflammation. We aimed to elucidate the biosynthesis and metabolism of 15dPGJ_2_ via conjugation with GSH, to form 15dPGJ_2_-glutathione (15dPGJ_2_-GS) and 15dPGJ_2_-cysteine (15dPGJ_2_-Cys) conjugates and to characterize the effects of mPGES-1 inhibition on the PGD_2_/15dPGJ_2_ pathway in mouse and human immune cells. Our results demonstrate the formation of PGD_2_, 15dPGJ_2_, 15dPGJ_2_-GS, and 15dPGJ_2_-Cys in RAW264.7 cells after lipopolysaccharide stimulation. Moreover, 15dPGJ_2_-Cys was found in lipopolysaccharide-activated primary murine macrophages as well as in human mast cells following stimulation of the IgE-receptor. Our results also suggest that the microsomal glutathione S-transferase 3 is essential for the formation of 15dPGJ_2_ conjugates. In contrast to inhibition of cyclooxygenase, which leads to blockage of the PGD_2_/15dPGJ_2_ pathway, we found that inhibition of mPGES-1 preserves PGD_2_ and its metabolites. Collectively, this study highlights the formation of 15dPGJ_2_-GS and 15dPGJ_2_-Cys in mouse and human immune cells, the involvement of microsomal glutathione S-transferase 3 in their biosynthesis, and their unchanged formation following inhibition of mPGES-1. The results encourage further research on their roles as bioactive lipid mediators.

There is growing evidence that selective microsomal prostaglandin E synthase-1 (mPGES-1) inhibitors represent an alternative therapeutic strategy to inhibit proinflammatory prostaglandin E2 (PGE_2_) production. Unlike nonsteroidal anti-inflammatory drugs, which target cyclooxygenases (COX) and suppress all downstream prostaglandins, selective mPGES-1 inhibition prevents the formation of induced PGE_2_ biosynthesis but spares other prostaglandins and may also lead to increased biosynthesis of anti-inflammatory arachidonic acid metabolites. The resulting changes in prostaglandin profiles observed in various cell types including cancer cells, fibroblasts, macrophages, and vessels raise the possibility of cardiovascular protection and enhanced anti-inflammatory effects following mPGES-1 inhibition (1, 2, 3, 4, 5, 6, 7). Previously, we showed that the eicosanoid profile is altered in lipopolysaccharides (LPS)-treated peritoneal macrophages from mPGES-1 KO mice compared with cells from WT mice. Specifically, levels of the prostaglandin D2 (PGD_2_) metabolite 15-deoxy-Δ^12,14^-prostaglandin J_2_ (15dPGJ_2_) were increased after deletion of mPGES-1 (8). PGD_2_ and 15dPGJ_2_ are both synthesized during the inflammatory response in the inflammatory exudates of mice with experimental peritonitis and contribute to the resolution of inflammation (9). In addition, 15dPGJ_2_ has been suggested to play a role in polarizing macrophages toward the anti-inflammatory M2 phenotype (10) and controlling the resolution phase of inflammation by inducing apoptosis in activated macrophages (11). Moreover, mPGES-1 was shown to regulate macrophage polarization toward a proinflammatory phenotype, whereas deletion of mPGES-1 resulted in upregulation of anti-inflammatory gene expression in macrophages (12). Furthermore, deletion of mPGES-1 as well as treatment with 15dPGJ_2_ protected against influenza A infection in rodents (13, 14). The extent to which inhibition of mPGES-1 might contribute to activation of the PGD_2_/15dPGJ_2_ pathway and macrophage polarization remains unclear.

15dPGJ_2_ is a bioactive product of PGD_2_ metabolism. PGD_2_, produced by COX and the hematopoietic PGD synthase during innate and adaptive immune responses can be further metabolized to 15dPGD_2_ and PGJ_2_ (9). The latter gives rise to two downstream cyclopentenone-prostaglandin metabolites of the J series, namely Δ^12^-PGJ_2_ and 15dPGJ_2_ (15). Among the PGD_2_ metabolites, 15dPGJ_2_ is the best studied and has gained increasing interest since its initial description in 1983 (16). 15dPGJ_2_ has been shown to function as a ligand of PPARγ (17, 18) and is linked to a variety of anti-inflammatory, anti-proliferative, cytoprotective, and proresolving activities (19, 20, 21). However, its physiological role has been questioned because only very low levels of free 15dPGJ_2_ have been detected *in vivo*, which contrasts with its activation of PPARγ *in vitro* at micromolar concentrations (22). Cyclopentenone prostaglandins are reactive lipid electrophiles that contain functional groups that can bind rapidly to the cysteine-containing tripeptide GSH and thiol groups in proteins such as Keap1, NF-_*К*_B, or HIF1α via Michael addition (23, 24). The electrophilic center at C9 within the cyclopentenone ring of 15dPGJ_2_ has been characterized as the primary site of Michael addition with GSH and proteins (25, 26). Glutathionylation of a variety of electrophiles has been described *in vitro* and *in vivo* where GSH conjugation can protect the cell from the accumulation of harmful electrophiles but also leads to the formation of bioactive metabolites such as the cysteinyl leukotrienes (LTC_4_, LTD_4_, LTE_4_) (27). Similar to the biosynthesis of the four-series leukotrienes, 15dPGJ_2_ has been shown to be metabolized in cell culture via the conjugation to GSH (25, 28). In 15dPGJ_2_-treated HepG2 cells, 15dPGJ_2_-glutathione (15dPGJ_2_-GS) has been described to be further metabolized via reduction at the cyclopentenone ring, removal of glutamic acid and glycine into a cysteine-conjugate (15dPGJ_2_-Cys) within 24 h (28). However, descriptions of their biosynthetic formation in immune cells are lacking and the function of these metabolites remains unclear. The cyclopentenone reactivity might be a reasonable explanation for the difficulty in detecting and quantifying free 15dPGJ_2_ in biological samples which challenged its role as an endogenous mediator.

The pleiotropic mechanisms that follow the inhibition of mPGES-1 lead to a decrease in proinflammatory PGE_2_ and as discussed here, to an enhancement of the PGD_2_/15dPGJ_2_ pathway. Thus, inhibition of mPGES-1, which is associated with anti-inflammatory and proresolving effects, holds great therapeutic potential and requires a detailed understanding of these pathways. Therefore, in this study, we investigated the biosynthesis of PGD_2_ metabolites, namely 15dPGJ_2_, 15dPGJ_2_-GS, and 15dPGJ_2_-Cys in mouse and human immune cells and examined the effects of mPGES-1 inhibition on the formation of these metabolites.

## Material and Methods

### Materials

Prostaglandins including 15-deoxy-Δ^12,14^-PGJ_2_ (CAY-18570-1) and 15-deoxy-Δ^12,14^-PGJ_2_-GS (CAY-18580-100) were purchased from Cayman Chemicals Co. (Ann Arbor MI). LPS from *Escherichia coli* (126M4087V) and reduced GSH were purchased from Sigma Aldrich (Germany). Recombinant murine Macrophage Colony-Stimulating Factor (M-CSF) (0914245) was purchased from Peprotech (Germany). 1 x RBC lysis buffer (4314838) was obtained from eBioscience. The COX inhibitors NS-398 and Diclofenac were purchased from Merck KGaA, Darmstadt, Germany and Sigma Aldrich (Germany), respectively. Selective mPGES-1 inhibitors compound III and compound 118 were described previously in (3, 29) and were produced by Gesynta pharma AB, Stockholm, Sweden. Inhibitors were reconstituted in DMSO to be used in experiments, whereby DMSO concentration did not exceed 0.05% in cell culture.

### *In vitro* preparation of 15dPGJ_2_-Cys conjugates with reduced carbonyl at position C11

15dPGJ_2_-Cys was prepared in 1 ml PBS containing 0.5 mg/ml L-Cysteine (Sigma), 1 mg GSH transferase from equine liver (Sigma), and 100 μg 15dPGJ_2_ (Cayman Chemical) at 37°C on a thermoblock, shaking for 2 h. The addition of glutathione-S-transferase (GST) to the 15dPGJ_2_-L-Cysteine mixture has been described previously (28) but was found to be expandable in order to achieve full conjugation. The reaction was stopped by addition of formic acid to a final concentration of 0.3% and the sample was subsequently loaded on a polymeric reverse phase sorbent column (StrataX-C18, Phenomenex). After the sample was loaded, the cartridge was rinsed with 1 ml 0.1% formic acid/ 5% methanol and the sample was eluted with 0.1% formic acid in methanol. Eluates were dried in a vacuum concentrator and the product obtained was dissolved in 0.5 ml methanol containing 1 mg/ml CeCl_3_^.^H_2_O and an aqueous solution of NaBH_4_ (12% w/w). The reduction reaction from a carbonyl to a hydroxyl at position C11 was carried out on ice for 1 h. Subsequently, the sample was acidified and applied to a polymeric reverse phase sorbent column and cleaned as described above. The eluate was dried and stored at −20°C until LC-MS/MS analysis. Conjugation quality and efficiency was assessed by LC-MS/MS analysis and full conjugation was assumed when no nonconjugated 15dPGJ_2_ could be detected.

### Plasma stability and pharmacokinetic analysis of 15dPGJ_2_-Cys

Plasma from freshly drawn heparinized (25 IU/ml) blood of healthy volunteers (in accordance with the Declaration of Helsinki and approved by the Regional Ethics Committee, Karolinska University Hospital, Sweden Dnr. 02-196) was obtained by centrifugation at 3000 *g* for 10 min. PGD_2_, 15d-PGJ_2_, 15d-PGJ_2_-GS, and 15d-PGJ_2_-Cys were prepared in DMSO and spiked into 100 μl plasma at a final concentration of 0.5 μM in a 96-well plate and incubated at 37°C for 0, 1, 3, 24, and 48 h. The samples were collected after incubation and stored at -20°C until preparation for LC-MS/MS analysis. Plasma samples were thawed on ice, spiked with 400 μl of 100% methanol containing the deuterated internal standards 6-keto-PGF_1α_-d4, PGF_2α_-d4, PGE_2_-d4, PGD_2_-d4, TxB_2_-d4, and 15-deoxy-Δ^12,14^-PGJ_2_-d4 (Cayman Chemical), followed by vortexing and centrifugation at 3000 *g* for 10 min at 4°C. Supernatants were collected and evaporated until complete dryness. Samples were reconstituted in 0.05% formic acid and subjected to solid phase extraction as described below.

For the pharmacokinetic analysis of 15dPGJ_2-_Cys, mice (n = 3 per time-point) were injected subcutaneously with 200 μl in-house generated 15dPGJ_2_-Cys (dissolved in 0.1% DMSO in saline solution to reach a final concentration of 5 μg/mouse). Blood (∼200 μl) was collected after 0.5, 1, and 2 h from the left ventricle using EDTA-collection tubes (BD Vacutainer®, 367841), while the mice were anesthetized by isoflurane (Sigma-Aldrich, Y0000858). Blood samples were stored at 4°C until centrifugation at 12,600 *g* at 4°C, followed by addition of methanol (600 μl). Protein precipitation and solid phase extraction were performed as described above and in the following. Animal experiments were approved by the regional Ethics Committee, Stockholm (Dnr. 7886-2018).

### Cloning, expression, and purification of MAPEG proteins

The various membrane associated proteins involved in eicosanoid and glutathione metabolism (MAPEG) protein genes were cloned into pPICZA yeast vector (Invitrogen) with six histidine-tags at N terminus as described earlier (30, 31). In brief, all plasmids were transformed into competent cells of the *Pichia pastoris KM71H* strain using *Pichia* Easy Comp Transformation kit (Invitrogen). The transformed yeast cells were cultivated in baffled flasks containing 2∗2 L minimal yeast medium containing glycerol at 27°C at 110 rpm shaking. The cells were harvested and again resuspended in 2 L of fresh minimal yeast medium supplemented with 0.6% (v/v) methanol every 24 h. The pH of the yeast medium was monitored and adjusted to 6.5 using 8% (v/v) NH3. Cells were harvested after 48 h and resuspended in breaking buffer (50 mM Tris-HCl pH 7.8, 100 mM KCl, 10% (v/v) glycerol). Cells were lysed by homogenization with glass beads in a Bead-Beater ((BioSpec Products, Inc.) six times with 5 min intervals at 4°C. Then, broken cells were filtered through nylon net and centrifuged at 1,500 g for 10 min to remove cell debris. The supernatant was solubilized in 1% (w/v) Triton X-100, 0.5% (w/v) sodium deoxycholate and 5 mM β-mercaptoethanol under constant stirring at 4°C for 1 h and further centrifuged at 10,000 *g* for 15 min at 4°C. The clear supernatant was passed through a Ni-Sepharose Fast Flow (GE Healthcare) column pre-equilibrated with 25 mM Tris/HCl pH 7.8,150 mM NaCl, 15 mM imidazole pH 7.8. The column was then washed with 15 volumes (CV) of wash buffer (25 mM Tris/HCl pH 7.8, 300 mM NaCl, 5% (v/v) glycerol, 5 mM β-mercaptoethanol, 0.03% (w/v) n-dodecyl β-D-maltoside (DDM), and 50 mM imidazole, pH 7.8) and protein was eluted with the same buffer containing 400 mM imidazole, pH 7.8. Pooled fractions were passed twice through S-hexylglutathione agarose column (Abcam and GE Healthcare). After passing the supernatant, the column was washed with buffer containing 25 mM Tris-HCl pH 8.0, 500 mM NaCl, 10% (v/v) glycerol, 5 mM β-mercaptoethanol, 0.03% DDM. Protein was eluted with and the same buffer containing 30 mM probenecid. The eluted protein was concentrated using amicon Ultra 50-kDa cutoff membrane (Millipore) and loaded on size exclusion chromatography with Superdex 200, 16/600 (GE Healthcare) column equilibrated with 25 mM Tris/HCl pH 7.5,150 mM NaCl, 5% (v/v) glycerol, 0.1 mM tris (2-carboxyethyl) phosphine, 0.03% DDM. Fractions containing the respective protein were combined and used directly for kinetics measurements. In some cases, protein was concentrated up to 0.8–1.0 mg/ml with 100 kDa cut off membrane (Millipore). Proteins were aliquoted, flash frozen in liquid nitrogen, and stored at -80°C until further use.

### Enzyme activity measurements of MAPEG proteins

Enzymatic formation of 15dPGJ_2_-conjugates by microsomal glutathione S-transferase 1 (MGST1), microsomal glutathione S-transferase 2 (MGST2), microsomal glutathione S-transferase 3 (MGST3), leukotriene C4 synthase (LTC4S), mPGES-1, and 5-lipoxygenase activating protein (FLAP) was tested. Briefly, 5 μg of each protein was added to 95 μl reaction buffer (25 mM Tris/HCl, pH 7.5, 100 mM NaCl, 0.03% DDM), containing reduced GSH (0.06 mM). The reaction was started when 15dPGJ_2_ (1 μg) was added and incubation continued for 5 min with gentle shaking. All reactions were performed in triplicates. After incubation for 5 min, the reactions were stopped with 800 μl 0.5% formic acid and reaction tubes were immediately placed on ice until lipid extraction. Control reactions without supplementing enzyme or substrate (15dPGJ_2_) were carried out in parallel. Assay conditions were optimized for incubation times (0.25 min–60 min) and GSH concentrations (0.06 mM–1 mM). Samples were then spiked with 50 μl deuterated internal standard containing 6-keto-PGF_1α_-d4, PGF_2α_-d4, PGE_2_-d4, PGD_2_-d4, TxB_2_-d4, and 15-deoxy-Δ^12,14^-PGJ_2_-d4 (Cayman Chemical) in 100% methanol and loaded on a preactivated and equilibrated Oasis-HLB 1 cc 30 mg cartridge (Waters), washed with 5% methanol, containing 0.05% formic acid and subsequently lipids were eluted in 100% methanol. Samples were evaporated and stored at -20ºC until LC-MS/MS analysis of the formation of 15dPGJ_2_-GS. Experiments with the soluble Mu class GST (Human, GSTM4-780H from Creative Bio Mart) were carried out under similar conditions. Assay conditions were varied for incubation times (up to 60 min) and protein concentrations (up to 10 μg per reaction). The MGST3 protein was tested in parallel as reference.

### *Km* determination of MGST3 protein

For determination of the Michaelis constant (*Km*) of MGST3, 5 μg enzyme was added to 95 μl reaction buffer (25 mM Tris-HCL, pH 7.5, 100 mM NaCl, 0.03% DDM), containing reduced GSH (0.1 mM). The reaction was started when 15dPGJ_2_ (1 μg–90 μg) was added and incubation continued for 5 min with gentle shaking at room temperature. Control reactions without supplementing enzyme or substrate (15dPGJ_2_) were carried out in parallel under similar conditions. All reactions were performed in triplicates. After incubation for 5 min at room temperature, the reactions were stopped with 200 μl 0.5% formic acid and reaction tubes were placed on ice immediately until analysis by HPLC. The enzymatic conjugation of 15dPGJ_2_ with GSH was monitored with a UV detector at 306 nm with 1:1:0.003 (v/v) acetonitrile/MilliQ water (MQ water)/acetic acid as mobile phase. Area units for 15dPGJ_2_-GS were collected, subtracted from background, and quantified based on spiked prostaglandin B_2_ (PGB_2_, 560 pmol, monitored as internal standard) with a correction coefficient of 0.4 (extinction coefficient 15dPGJ_2_ (12,000) / extinction coefficient of PGB_2_ (30,000)). Data were expressed as pmol/μg/min and the *Km* value was calculated using hyperbolic regression analysis.

### Macrophage cell line culture

The murine macrophage cell line RAW264.7 (ATCC) was cultured in DMEM supplemented with 10% fetal bovine serum (FBS), 100 U/ml penicillin, 0.1 mg/ml streptomycin, and 1 mM sodium pyruvate and 2 mM L-glutamine at 37°C in a humidified 5% CO_2_ atmosphere. Passaging of cells was performed using PBS-EDTA (5 mM) solution and passages 5–10 were used for experiments. For stimulation, cells were plated in 24-well plates, 12-well plates, or 6-well plates at concentrations of 6–10 × 10^4^ cells/cm^2^ if not indicated differently. Cells were allowed to adhere for 24 h, then medium was aspirated and replaced with fresh culture medium containing 2 μg/ml LPS and respective treatments (NS-398, 0.1 μM; CIII, 10 μM; 118, 1 μM) for various time points. For transcription and translation inhibition, RAW264.7 cells were pretreated for 4 h with 5 μg/ml actinomycin D (Cayman Chemical, 11421) or 100 μg/ml cycloheximide (Cayman chemical, 14126) prior addition of 2 μM 15dPGJ_2_ for various time points.

### Primary macrophage cell culture

WT and mPGES-1 KO mice were on an inbred DBA/1lacJ genetic background and generated by breeding heterozygous littermates as described previously (32). All animal experiments were approved by the regional Ethics Committee, Stockholm (Dnr. 7886-2018). For the preparation of bone marrow-derived macrophages (BMDMs), mice were sacrificed by CO_2_ inhalation, and femoral and tibia bones from hind legs were dissected and cleaned from all remaining tissue. Bone ends were cut open and bone marrow was flushed out. The bone marrow suspension was filtered through a 70 μm cell strainer; red blood cells were lysed with 1 × RBC lysis buffer and bone marrow cells were subsequently reconstituted in DMEM cell culture medium supplemented with 10% FBS, 100 U/ml penicillin, 0.1 mg/ml streptomycin, and 1 mM sodium pyruvate, 2 mM L-glutamine, and 2 mM Hepes in the presence of 20 ng/ml M-CSF. Cells were cultured in low attachment cell culture flasks at 37°C in a humidified 5% CO_2_ atmosphere for six days. After three days, half of the cell culture medium was exchanged with fresh culture medium supplemented with 40 ng/ml M-CSF and incubation was continued. After six days in culture, the medium was aspirated and cells were detached from the cell culture flasks using PBS-EDTA (5 mM), counted, and plated at various densities for stimulation experiments.

For macrophage activation, cells were stimulated with 2 μg/ml LPS if not indicated otherwise. When cells were co-incubated with GSH, 0.5 mM GSH in PBS was supplemented to the culture medium. For kinetic experiments in Figs. 2 and 4, BMDMs were directly seeded to 24-well plates, differentiated for six days, and then treated. In these experiments, prostanoid levels were normalized to protein concentrations. Protein concentration determination of cell pellets after lysis (50 mM Tris-HCl, 150 mM NaCl, 1% SDS, pH 7.9) and sonication for 15 min was measured by the BCA (Thermo Fisher) assay according to manufacturer’s instructions.

### Primary human mast cell culture

For the generation of cord-blood derived mast cells (CBMCs), CD34^+^-hematopoietic progenitors were isolated from cord-blood and cultured as previously described (33), (in accordance with the Declaration of Helsinki and approved by the regional Ethics Committee, Stockholm, Dnr. 2019-01729,). When the cells reached about 90% tryptase positivity, they were activated. Prior anti-IgE-activation, the cells were treated with 10 ng/ml human recombinant IL-4 (PeproTech) for four days, and the day before activation, 1 μg/ml human IgE (Calbiochem) was added. Cells were plated to 24-well plates at a density of 0.5 × 10^6^ cells/ml or 1 × 10^6^ cells/ml, and the cells were stimulated with 2 μg/ml anti-IgE (Sigma) with or without 118 (1 μM) or diclofenac (1 μM) or only culture medium for unstimulated controls and incubated for 24 h at 37°C. Subsequently, cells and supernatants were transferred to reaction tubes, centrifuged at 300 g for 10 min, and supernatants were collected and frozen at −20°C until LC-MS/MS analysis of lipid mediators. Cells were collected and frozen at −80°C until RNA extraction.

### Generation of RAW264.7 cells lacking MGST3

The CRISP-Cas9 system was used to generate MGST3 KO cells. The sgRNA was designed using the Green Listed software (http://greenlisted.cmm.ki.se/) (34) and the Brie reference library (35) selecting the sgRNA with the highest on-target score. A 2′-O-methyl modified and phosphorothioate stabilized version of the sgRNA (Mgst3: CGACACTCACGTGTTCTGGT) was ordered from Sigma-Aldrich. For sequencing, forward primer: ACCAATGCCCTCGTTCACAT and reverse primer: CAGAAAACCAGGCGCTCAT were designed to generate a 500–700 bp amplicon with the sgRNA binding site in the middle of the amplicon.

RAW264.7 cells were cultured in DMEM supplemented with 10% FBS and penicillin-streptomycin. The Neon Electroporation system was used to deliver CRISPR components according to manufacturer suggestions (pulse voltage: 1,680, pulse width: 20 ms, and pulse number: 1). CRISPR reaction with ∼500 pmol sgRNA and 20 μM spCas9 (Sigma-Aldrich) were delivered into 5 × 10^6^ cells using the Neon 10 μl kit. Single cell clones were generated and screened for complete knock out of the gene. Typically, cells were harvested for PCR amplicon generation and sequenced using Sanger sequencing and analyzed by ICE (https://ice.synthego.com) for indels. Cells with >90% of indel were used for further experiments.

### Lipid extraction

Supernatants were thawed on ice, acidified with 0.5% formic acid, and spiked with 50 μl deuterated internal standard containing 6-keto-PGF_1α_-d4, PGF_2α_-d4, PGE_2_-d4, PGD_2_-d4, TxB_2_-d4, and 15-deoxy-Δ^12,14^-PGJ_2_-d4 (Cayman Chemical) in 100% methanol. Samples were loaded on a preactivated and equilibrated Oasis-HLB 1 cc 30 mg cartridge (Waters), washed with 5% methanol, containing 0.05% formic acid, and subsequently lipids were eluted in 100% methanol. Samples were evaporated and stored at −20°C until LC-MS/MS analysis.

Cell pellets were thawed on ice and resuspended in 400 μl ice cold 100% methanol spiked with 50 μl deuterated internal standard containing 6-keto-PGF_1α_-d4, PGF_2α_-d4, PGE_2_-d4, PGD_2_-d4, TXB_2_-d4, and 15-deoxy-Δ^12,14^-PGJ_2_-d4 (Cayman Chemical). Samples were mixed (10 × by pipetting) and vortexed prior to a 20 min incubation on ice. Thereafter, samples were centrifuged (9,000 *g*, 10 min at 4°C) and supernatants were transferred to a fresh reaction tube. Remaining pellets were suspended with 100 μl ice cold 100% methanol, mixed, vortexed, and subsequently centrifuged (9,000 *g*, 10 min at 4°C). Supernatants were transferred to already collected supernatants. Samples were evaporated and reconstituted in 50 μl of 20% acetonitrile/MQ water and incubated at 4°C for 20 min. Samples were centrifuged (13,000 *g*, 10 min at 4°C) and supernatants were transferred to injection vials for LC-MS/MS analysis.

### LC-MS/MS analysis of prostanoids

Samples were reconstituted in 50 μl of 20% acetonitrile/MQ and separated on a 50 × 2.1 mm Acquity UPLC BEH C18, 1.7 μm column (Waters) in a 13 min linear gradient with 0.05% formic acid/MQ water as mobile phase A and 0.05% formic acid/acetonitrile as mobile phase B at a flow rate of 0.6 ml/min. Analytes were quantified by multiple reaction monitoring (MRM) in negative mode for all prostanoids as described previously (8), except of the 15dPGJ_2_-conjugates which were primarily analyzed in positive mode (supplemental Table S1), using a triple quadrupole mass spectrometer (Acquity TQ detector, Waters). Data presented in Figs. 2E and S2E were acquired on an Acquity Xevo TQ-XS UPLC/MS system (Waters) with analyte separation on a 50 × 2.1 mm Acquity UPLC BEH C18, 1.7 μm column (Waters) in a 17 min linear gradient with 0.05% formic acid/MQ water as mobile phase A and 0.05% FA/10% isopropanol in acetonitrile as mobile phase B at a flow rate of 0.5 ml/min. MRM transitions applied were *m/z* 624.0 > 308.0 (ES+) for 15dPGJ_2_-GS and *m/z* 438.0>351.0 for 15dPGJ_2_-Cys (ES-). Raw data were processed and analyzed using MassLynx software (version 4.1 and 4.2) and quantified against external standard curve with internal standard calibration for 6-keto-PGF_1α_, PGF_2α_, PGE_2_, PGD_2_, TXB_2_, and 15-deoxy-Δ^12,14^-PGJ_2_. For the quantification of 15dPGJ_2_-GS and 15dPGJ_2_-Cys, standard calibration curves were generated with the commercial standard or in-house generated metabolite, respectively, at serial concentrations ranging from 0 to 48 pmol injected. Deuterated internal standard was not available for these conjugates. Peak areas were recorded and the concentration of the metabolites in unknown samples were determined using the obtained calibration curves for each metabolite, respectively.

### Quantitative RT-PCR for MGST3

Cells were cultured and treated as described earlier. After 24 h, the cells were washed once with PBS and lysed (RLT lysis buffer, QIAGEN) in the culture vessel according to manufacturer’s instructions. Cell culture plates were subsequently frozen at -80°C until RNA extraction. RNA was isolated following manufacturer’s instructions using the RNeasy Plus Mini Kit (250) from QIAGEN and mRNA concentrations were measured with a Nanodrop spectrophotometer (Thermo Fischer). Subsequently, 0.5–1 μg template RNA was reverse transcribed into cDNA using the SuperScript™ Vilo™ cDNA synthesis kit (Thermo Fisher). Prior to reverse transcription of the extracted RNA from human mast cells, heparinase treatment was performed. RNA (1 μg) was incubated for 2 h at 25°C in 5 mM Tris-HCl pH 7.5, 1 mM CaCl_2_ (0.1% BSA) with 2.5 U heparinase (Sigma Aldrich), and 2 U RNase inhibitor (Ambion).

Quantitative real-time PCR was performed on ABI 7300 Real-Time PCR system using the TaqMan™ Gene Expression Master Mix (# 4369514, Thermo Fisher) and TaqMan™ Gene Expression assays for the target genes mouse *Mgst2* (Mm00723390_m1), mouse *Mgst3* (Mm00787806_s1), and mouse *Ltc4s* (Mm00521864_m1). The relative mRNA expression was quantified by the ΔΔCt method comparing treated samples to unstimulated controls after normalizing with the endogenous control gene β-actin *Actb* (Mm00607939_s1). For the target gene analysis in human mast cells, the TaqMan™ Gene Expression Assays for human *MGST2* (Hs00992727_g1), human *MGST3* (Hs01058946_m1), human *LTC4S* (Hs00168529_m1), and human β-actin *ACTB* (Hs99999903_m1) were used.

### Statistical analysis

Results are expressed as mean ± standard deviation of n independent experiments or n number of animals or donors. Calculations and graphs were prepared using GraphPad Prism version 9.0 (GraphPad software Inc.). Comparisons between two groups were performed using unpaired two-tailed student’s *t*-test. Statistical significance level was indicated as ∗ *P* < 0.05.

## Results

### LC-MS/MS method for the detection of 15dPGJ_2_-GS and 15dPGJ_2_-Cys

**Fig. 1:**
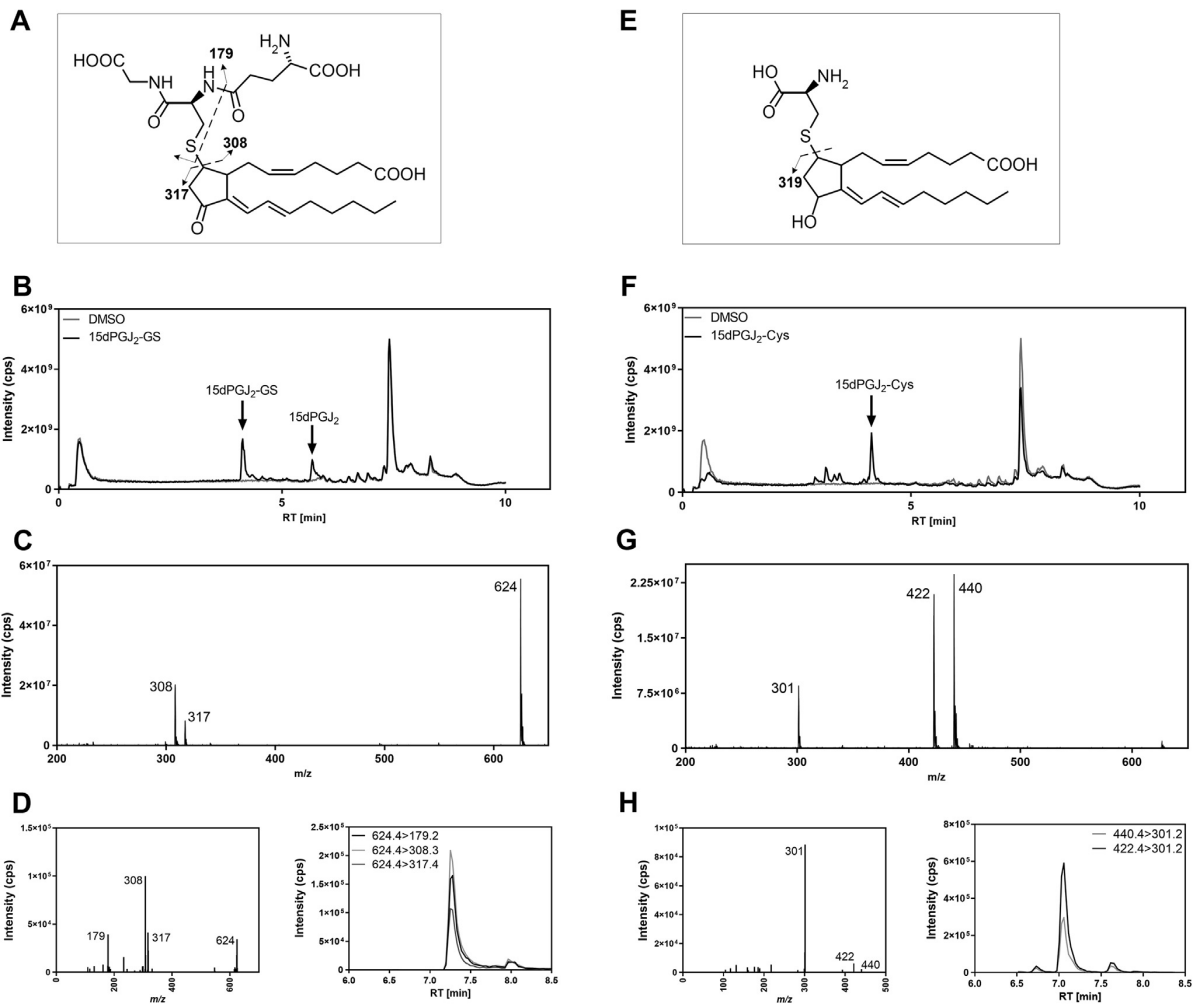
Analysis of 15dPGJ_2_ conjugates by LC-MS/MS. Structures for 15dPGJ_2_-GS (A) and 15dPGJ_2_-Cys (E). Full scan total ion chromatogram (*m/z* 200–650) for the commercial 15dPGJ_2_-GS standard (B) eluting at 4.2 min and for 15dPGJ_2_-Cys (F) eluting at 4.0 min. Mass spectra (*m/z* 200–650) for 15dPGJ_2_-GS (C) and for 15dPGJ_2_-Cys (G). D: Fragmentation spectra (*m/z* 100–630) of precursor ion *m/z* 624 (left panel) and representative multiple reaction monitoring (MRM) chromatograms (right panel) for 15dPGJ_2_-GS with the fragments *m/z* 308, *m/z* 317, and *m/z* 179. H: Fragmentation spectra (*m/z* 100–445) of precursor ion *m/z* 440 (left panel) and representative multiple reaction monitoring chromatograms (right panel) for 15dPGJ_2_-Cys with the precursor mass *m/z* 440 and *m/z* 422 and the fragment *m/z* 301. The intensity corresponds to counts per second (cps) measured. 15dPGJ_2_, 15-deoxy-Δ12,14-prostaglandin J_2_; 15dPGJ_2_-GS, 15dPGJ_2_-glutathione; 15dPGJ_2_-Cys, 15dPGJ_2_-cysteine.

To study the metabolism of 15dPGJ_2_ via the conjugation to GSH, we set up a targeted LC-MS/MS method for the analysis of 15dPGJ_2_-GS and 15dPGJ_2_-Cys conjugates (Fig. 1 and supplemental Table S1). Compounds were separated in a 10 min linear gradient with 0.05% formic acid/MQ water as mobile phase A and 0.05% formic acid/acetonitrile as mobile phase B. Collection of the full scan total ion chromatogram (*m/z* 200–650) for the commercial 15dPGJ_2_-GS standard showed a major peak eluting at 4.2 min and *m/z* 624.4, corresponding to [M+H]^+^ of 15dPGJ_2_-GS (calculated formula weight 623.3) (Fig. 1B, C). The smaller peak at 7 min corresponds to unconjugated 15dPGJ_2_. The major fragments in a fragment spectrum of *m/z* 624.4 were *m/z* 308 [M-15dPGJ_2_+H]^+^ and *m/z* 317 [M+H-GSH]^+^, which represent GSH and 15dPGJ_2,_ respectively (Fig. 1D, left panel). The *m/z* 179 fragment represents the cysteine-glycine residue of GSH (Fig. 1D, left panel). Analysis for the precursor mass eluting at 4.0 min for 15dPGJ_2_-Cys [M+H]^+^ revealed the precursor ion at *m/z* 440.4 (calculated formula weight 439.2) (Fig. 1F, G). The fragmentation spectrum for *m/z* 440.4 represents the reduced conjugate *m/z* 440.4 and *m/z* 422 [M+H-H_2_O]^+^ as well as the free reduced 15dPGJ_2_ with *m/z* 301 which probably corresponds to loss of water (Fig. 1H, left panel). In a longer 13 min, analytical gradient 15dPGJ_2_-GS eluted at 7.25 min (Fig. 1D, right panel) and 15dPGJ_2_-Cys eluted at 7.14 min (Fig. 1H, right panel). The obtained MRM transitions for 15dPGJ_2_-GS were *m/z* 624.4>308.3, *m/z* 624.4>317.4, *m/z* 624.4>179.2; for 15dPGJ_2_-Cys, *m/z* 440.4>301.2, *m/z* 422.4>301.2; and for 15dPGJ_2_, *m/z* 315.1>271.1 was used. Extraction of prostaglandins including the 15dPGJ_2_-conjugates was performed by solid phase extraction. Losses due to matrix effect were 17% for 15dPGJ_2_-GS and 26% for 15dPGJ_2_-Cys compared to nonextracted standard. The recovery rate comparing internal standards spiked into cell culture medium matrix before and after solid phase extraction was about 100% for 15dPGJ_2_-GS and 86% for 15dPGJ_2_-Cys. The lower limit of quantification defined as signal to noise >10 was determined to 0.1 pmol in solution injected on column (supplemental Fig. S1). Analysis of synthetic standards for LT-C_4_ and LTE_4_ with the same *m/z* as the 15dPGJ_2_-metabolites showed clear separation in retention times (supplemental Fig. S1G). Stability measurements in plasma demonstrated superior stability of the 15dPGJ_2_-Cys conjugate up to 48 h compared to PGD_2_ which was reduced to 1% of its initial concentration after 24 h as well as 15dPGJ_2_ and 15dPGJ_2_-GS which were significantly diminished already after 3 h (supplemental Fig. S2). However, *in vivo* pharmacokinetics showed poor stability of the 15dPGJ_2_-Cys conjugate in mouse plasma within 1 h post injection (supplemental Fig. S2E).

### Metabolism of 15dPGJ_2_ into 15dPGJ_2_-GS and 15dPGJ_2_-Cys in RAW264.7 cells and BMDM

**Fig. 2:**
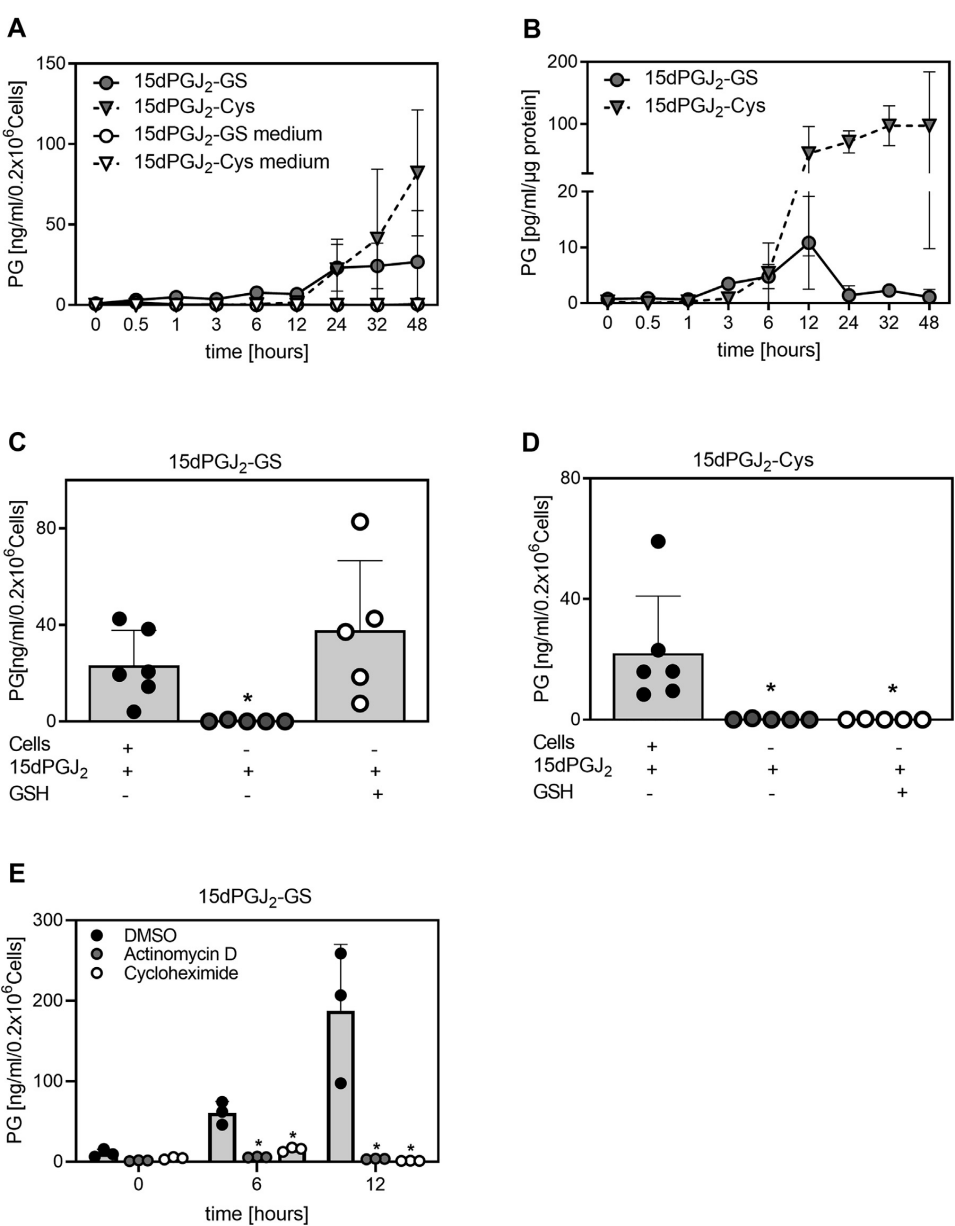
Analysis of 15dPGJ_2_ metabolism in macrophages treated with 15dPGJ_2_. A: Time-dependent formation of prostaglandin (PG)-metabolites in RAW264.7 cells or cell-free culture media. The data show 3–6 independent experiments. B: Time-dependent formation of 15dPGJ_2_-metabolites in BMDM from WT mice (n = 3). C, D, Cell-dependent formation of 15dPGJ_2_-GS and 15dPGJ_2_-Cys in the presence of RAW264.7 cells or cell-free culture media in the presence or absence of GSH after 24 h. The data show 5–6 independent experiments. ∗*P* < 0.05 indicates significant difference compared to cells incubated with 15dPGJ_2_. E: Time-dependent formation of 15dPGJ_2_-GS in RAW264.7 cells incubated with 15dPGJ_2_ (2 μM) and actinomycin D or cycloheximide. The data show three independent experiments. ∗*P* < 0.05 indicates significant difference compared to cells incubated with the DMSO control. 15dPGJ_2_, 15-deoxy-Δ12,14-prostaglandin J_2_; BMDM, bone marrow derived macrophages; 15dPGJ_2_-Cys, 15dPGJ_2_-cysteine; 15dPGJ_2_-GS, 15dPGJ_2_-glutathione.

To study the metabolism of exogenous 15dPGJ_2_ by mouse macrophages, RAW264.7 cells or BMDMs were incubated in the presence of 15dPGJ_2_ (2 μM) for 0, 0.5, 1, 3, 6, 12, 24, 32, and 48 h, and the supernatants were analyzed for the formation of 15dPGJ_2_ metabolites. No exogenous GSH was added to the culture medium in these experiments. In RAW264.7 cells, we observed the formation of 15dPGJ_2_-GS, which reached a plateau 24 h after treatment. Production of 15dPGJ_2_-Cys increased after 12 h, and levels continued to increase over time (Fig. 2A). Depletion of serum albumin did not reduce the formation of 15dPGJ_2_-conjugates, it rather accelerated the formation of 15dPGJ_2_-GS (data not shown). This could be because serum albumin could act as a carrier for fatty acids in extracellular fluids, it might trap 15dPGJ_2_ in an albumin-fatty acid complex or covalently bind 15dPGJ_2_ via the free –SH group in cysteine 34 of albumin delaying the cellular uptake (36, 37). Moreover, inhibition of transcription and translation with actinomycin D and cycloheximide blocked the formation of 15dPGJ_2_-GS (Fig. 2E). In BMDM treated with 15dPGJ_2_, the 15dPGJ_2_-GS conjugate increased after 3 h and peaked at 12 h, whereas the 15dPGJ_2_-Cys conjugate increased continuously after 6 h (Fig. 2B). In cell-free cell culture medium aspirated from untreated RAW264.7 cells and centrifuged prior to incubation with 15dPGJ_2_, we observed significantly reduced amounts of 15dPGJ_2_-GS and 15dPGJ_2_-Cys (Fig. 2A, C , D). Co-incubation of 15dPGJ_2_ with an excess of GSH in cell-free cell culture medium resulted in the formation of 15dPGJ_2_-GS to the same levels as in the presence of RAW264.7 cells, measured after 24 h. However, the addition of GSH did not result in the formation of equivalent levels of 15dPGJ_2_-Cys, which together with the observed inhibition of 15dPGJ_2_-GS after transcription and translation inhibition, suggests enzyme involvement in the metabolism of GSH-conjugates of 15dPGJ_2_.

### MGST3 is involved in 15dPGJ_2_-conjugate formation

**Fig. 3:**
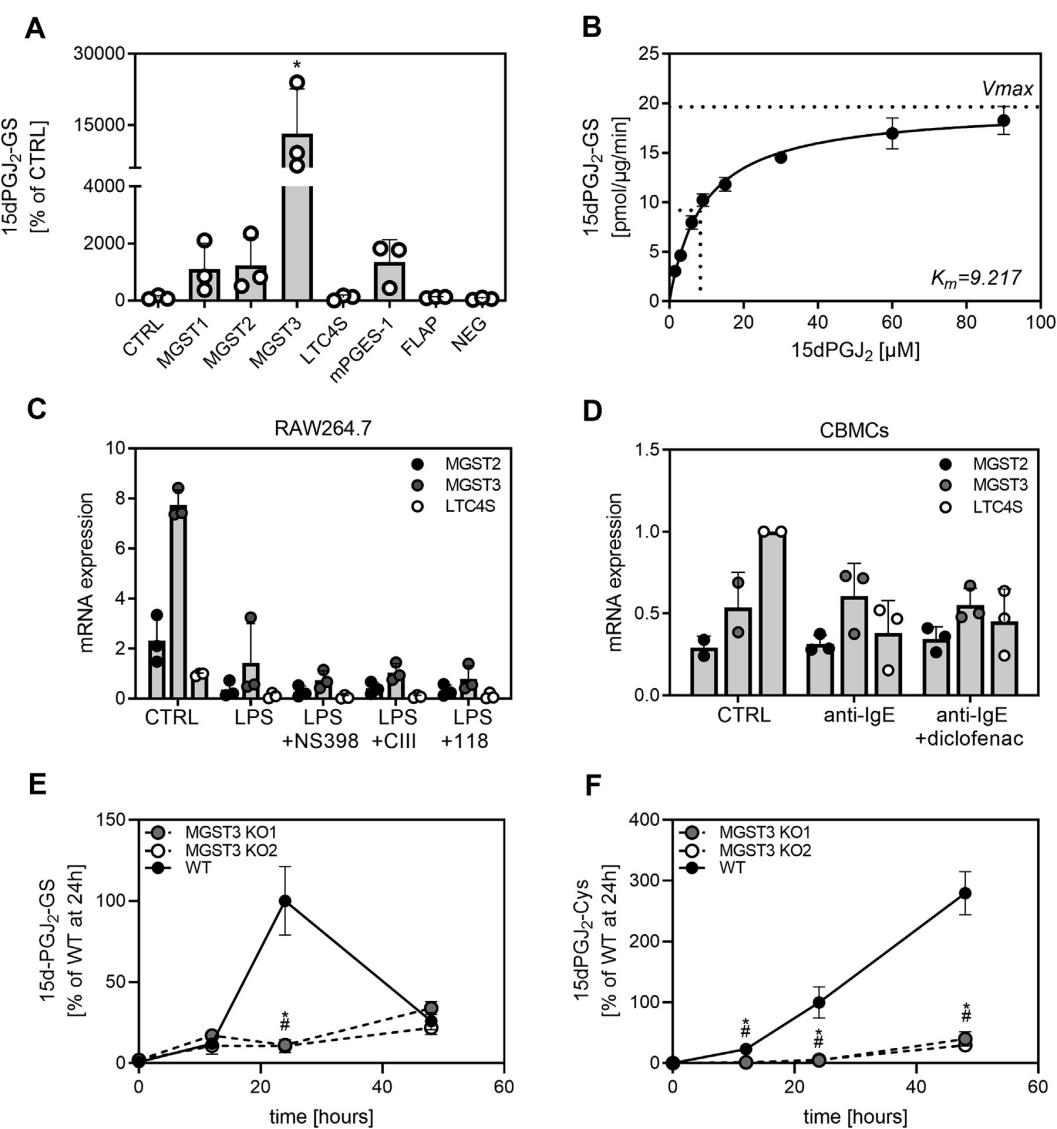
Conversion of 15dPGJ_2_ to 15dPGJ_2_-GS by enzymes of the MAPEG family. A: Enzymatic formation of 15dPGJ_2_-GS in the presence of MGST1, MGST2, MGST3, LTC4S, mPGES-1, and FLAP. Measurements of 15dPGJ_2_-GS after incubation of enzymes with the substrate 15dPGJ_2_ in the presence of excess GSH for 5 min by LC-MS/MS. The data show three independent experiments. Analytes were quantified using the MRM transition *m/z* 624.4>317.4. ∗*P* < 0.05 indicates significant difference to the control (CTRL, without MGST3 enzyme). Reactions with MGST3 but without substrate supplementation (15dPGJ_2_) were carried out in parallel and referred to as negative control (NEG). B: 15dPGJ_2_ (0.75–90 μM) was incubated in the presence of MGST3 (5 μg) and GSH (0.1 mM) for 5 min. Control reactions (CTRL) without MGST3 enzyme were carried out in parallel. Vmax and apparent *Km* were calculated using hyperbolic regression analysis after subtraction of background (CTRL incubations) and quantification to reference compound PGB_2_. The data show three independent experiments. Relative expression of MGST2, MGST3, and LTC4S were measured by qRT-PCR in (C) RAW264.7 cells and (D) CBMCs (cord-blood derived mast cells) after different treatments. Relative mRNA expression to LTC4 in control cells is presented. RAW264.7 cells were treated with LPS for 24 h in the presence or absence of the COX-2 inhibitor NS-398 (0.1 μM) and the mPGES-1 inhibitors CIII (10 μM) or 118 (1 μM). CBMCs were stimulated with anti-IgE for 24 h in combination with a COX-1/COX-2 inhibitor diclofenac (1 μM). In one experiment, mRNA levels in the control samples were below detection limit and therefore removed. β-actin was used as an internal control to normalize target gene expression. The data show three independent experiments. Formation of 15dPGJ_2_-GS (E) and 15dPGJ_2_-Cys (F) in supernatants of RAW264.7 cells lacking MGST3. RAW264.7 WT cells or RAW264.7 MGST3 KO cells (2 different clones were analyzed, referred to as MGST3 KO1 and MGST3 KO2) were incubated with 15dPGJ_2_ (2 μM) for 12–48 h. Data are expressed as percentage of levels measured at the 24 h time point and presented as triplicates. Significant difference of KO cells compared to WT cells is indicated (∗*P* < 0.05 for MGST3 KO1, ^#^*P* < 0.05 for MGST3 KO2). 15dPGJ_2_, 15-deoxy-Δ12,14-prostaglandin J_2_; 15dPGJ_2_-GS, 15dPGJ_2_-glutathione; 15dPGJ_2-_Cys, 15dPGJ2-cysteine; MAPEG, membrane associated proteins involved in eicosanoid and glutathione metabolism; COX, cyclooxygenase; MGST, microsomal glutathione S-tranferase; LTC4S, leukotriene C4 synthase; FLAP, 5-lipoxygenase activating protein; mPGES-1, microsomal prostaglandin E synthase-1; MRM, multiple reaction monitoring; LPS, lipopolysaccharide.

To study the possible involvement of GSTs in the metabolism of 15dPGJ_2_, we screened the enzyme activity of MGST1, MGST2, MGST3, LTC4S, FLAP, and mPGES-1, members of the MAPEG family for conjugation of 15dPGJ_2_ with GSH. We found that MGST3 significantly enhanced the formation of 15dPGJ_2_-GS after 5 min, compared with control reactions without enzyme. MGST1, MGST2, and mPGES-1 showed weak activity toward 15dPGJ_2_-GS conjugate formation, without reaching significance. LTC4S and FLAP had no effect on 15dPGJ_2_-GS formation (Fig. 3A). The apparent Michaelis constant (*Km*) for MGST3 was determined to 9.2 μM (Fig. 3B). Parallel measurements of 15dPGJ_2_-Cys indicated no direct activity of the MAPEG proteins towards generation of 15dPGJ_2_-Cys in the activity assay. In addition, a soluble GST of the Mu class was tested for the formation of 15dPGJ_2_-GS and 15dPGJ_2_-Cys under similar conditions. No enzyme activity was detected regarding production of both conjugates (supplemental Fig. S7, representative results for 15dPGJ_2_-GS), even after longer incubation times and higher protein amounts (data not shown).

To understand whether MGST3 might play a role in the metabolism of 15dPGJ_2_ *in vitro*, we analyzed MGST3 mRNA levels in RAW264.7 cells and primary human mast cells (CBMCs). We also assessed whether inhibition of mPGES-1 or COX affects MGST3 mRNA expression. In RAW264.7 cells, *Mgst3* expression was relatively high compared with *Mgst2* and *Ltc4s* regardless of the treatment (Fig. 3C). Treatment with the mPGES-1 inhibitors CIII and 118 or the COX-2 inhibitor NS-398 had no effect on *Mgst3* expression compared with the LPS control. Decreased *Mgst2*, *Mgst3*, and *Ltc4s* mRNA levels were observed upon LPS treatment compared with unstimulated control. In CBMCs, *MGST3* expression, similarly to *MGST2* and *LTC4S*, was not affected by anti-IgE stimulation and diclofenac treatment (Fig. 3D). Moreover, we found upregulation of *Mgst3* expression after treatment of RAW264.7 cells with 15dPGJ_2_ for 12 or 24 h (supplemental Fig. S3), which may serve as an explanation for the delayed formation of 15dPGJ_2_-conjugates in the kinetic experiments in Fig. 2 and the inhibition seen after actinomycin D and cycloheximide treatment.

We next tested the formation of 15dPGJ_2_-GS and 15dPGJ_2_-Cys in RAW264.7 cells lacking MGST3. RAW264.7 WT and MGST3 KO cells were treated with 15dPGJ_2_ (2 μM) for various time points, and the supernatants were analyzed for the formation of 15dPGJ_2_-GS and 15dPGJ_2_-Cys. The formation of both conjugates was significantly reduced in cells lacking MGST3, indicating that MGST3 is an essential enzyme in this metabolic pathway (Fig. 3E, F). The formation of 15dPGJ_2_-GS was significantly reduced in MGST3 KO cells compared with WT cells at 24 h. The formation of 15dPGJ_2_-Cys was significantly reduced in MGST3 KO cells compared with WT cells at 12 h, 24 h, and 48 h.

### Endogenous formation of the 15dPGJ_2_ metabolites in macrophages and mast cells

To identify endogenous 15dPGJ_2_-metabolites, we studied their production by the murine monocyte-macrophage cell line RAW264.7, primary murine macrophages, and primary human mast cells.

Analysis of cell supernatants and cells showed that the 15dPGJ_2_-GS and 15dPGJ_2_-Cys conjugates were endogenously produced by RAW264.7 cells upon LPS stimulation, whereby the supernatants showed higher levels of both conjugates than the cell pellets (supplemental Fig. S4).

**Fig. 4:**
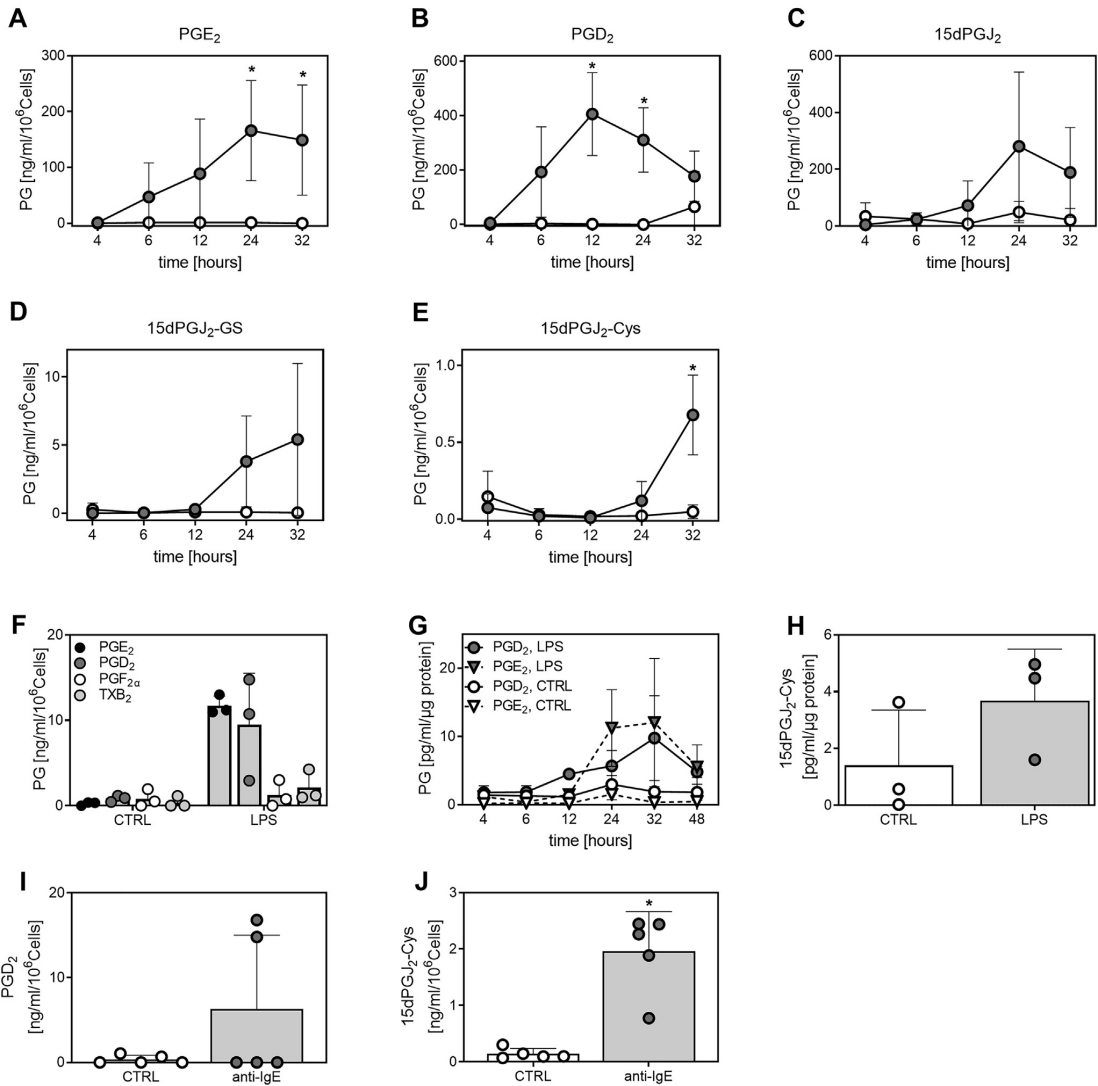
Endogenous production of PGD_2_ metabolites in RAW264.7 cells, BMDM, and mast cells. (A–E) RAW264.7 cells were treated with LPS (•) for 4, 6, 12, 24, or 32 h and PG levels in supernatants were compared to respective time controls (Control represents untreated cells, ○). Data show four independent experiments. ∗*P* < 0.05 indicates significant difference to the control. (F) Prostaglandins were analyzed in supernatants of LPS-treated BMDM from wild type mice after 24 h (n = 3). (G) PGE_2_ and PGD_2_ formation over time in BMDM from wild type mice (n = 3), CTRL represents untreated cells. (H) Quantification of 15dPGJ_2_-Cys (422.4>301.2) after 32 h, n = 3. (I, J) PGD_2_ (n = 5) and 15dPGJ_2_-Cys (422.4>301.2) (n = 5) were quantified in supernatants from CBMCs stimulated with anti-IgE for 24 h. In three donors, the PGD_2_ levels were below quantification limit (data points set to 0). ∗*P* < 0.05 indicates significant difference to the control (CTRL). CBMCs, cord-blood derived mast cells; BMDM, bone marrow derived macrophages; PGD_2,_ prostaglandin D_2_; LPS, lipopolysaccharides; 15dPGJ_2_-Cys, 15dPGJ_2_-cysteine.

To better understand the biosynthesis and metabolism of PGD_2_, we performed kinetic experiments in LPS-stimulated macrophages. LPS treatment of RAW264.7 cells revealed that PGD_2_ levels were highest after 12 h of incubation and declined thereafter (Fig. 4B), whereas PGE_2_ levels increased steadily until the 24 h time point (Fig. 4A). We found that PGD_2_ is the predominant prostaglandin produced by RAW264.7 cells with levels in the cell supernatants two-fold higher than those of PGE_2_. 15dPGJ_2_, 15dPGJ_2_-GS, 15dPGJ_2_-Cys, PGF_2α_, and low levels of TXB_2_ were also detected (Figs. 4 and S4). 15dPGJ_2_ and its GSH metabolites were formed and released at later time points starting at 12 h after treatment, with significant formation of 15dPGJ_2_-Cys after 32 h (Fig. 4C–E).

We next assessed the prostaglandin profile in LPS-treated BMDM from WT mice. LPS-treated BMDM produced primarily PGE_2_ and PGD_2_ as well as low levels of PGF_2a_ and TXB_2_ after 24 h (Figs. 4F and S5A). Female mice produced higher levels of prostanoids and were therefore used for further analysis (Figs. 4F and S5A). PGE_2_ production reached highest levels at 24 and 32 h, whereas PGD_2_ levels began to increase at 12 h and peaked at 32 h where we also identified 15dPGJ_2_-Cys (Fig. 4G, H). Levels of 15dPGJ_2_ and 15dPGJ_2_-GS were below quantification limit.

To identify endogenously formed 15dPGJ_2_-metabolites in human primary cells, we analyzed supernatants from human CBMCs. PGD_2_, 15dPGJ_2_, 15dPGJ_2_-GS, and 15dPGJ_2_-Cys were identified in anti-IgE stimulated cells, with levels of 15dPGJ_2_ and 15dPGJ_2_-GS below the limit of quantification. 15dPGJ_2_-Cys was significantly increased in response to stimulation with anti-IgE for 24 h (Figs. 4I, J and S6). No PGE_2_ was detected in the analyzed supernatants.

### Effects of mPGES-1 inhibition on the PGD_2_/15dPGJ_2_ pathway

**Fig. 5:**
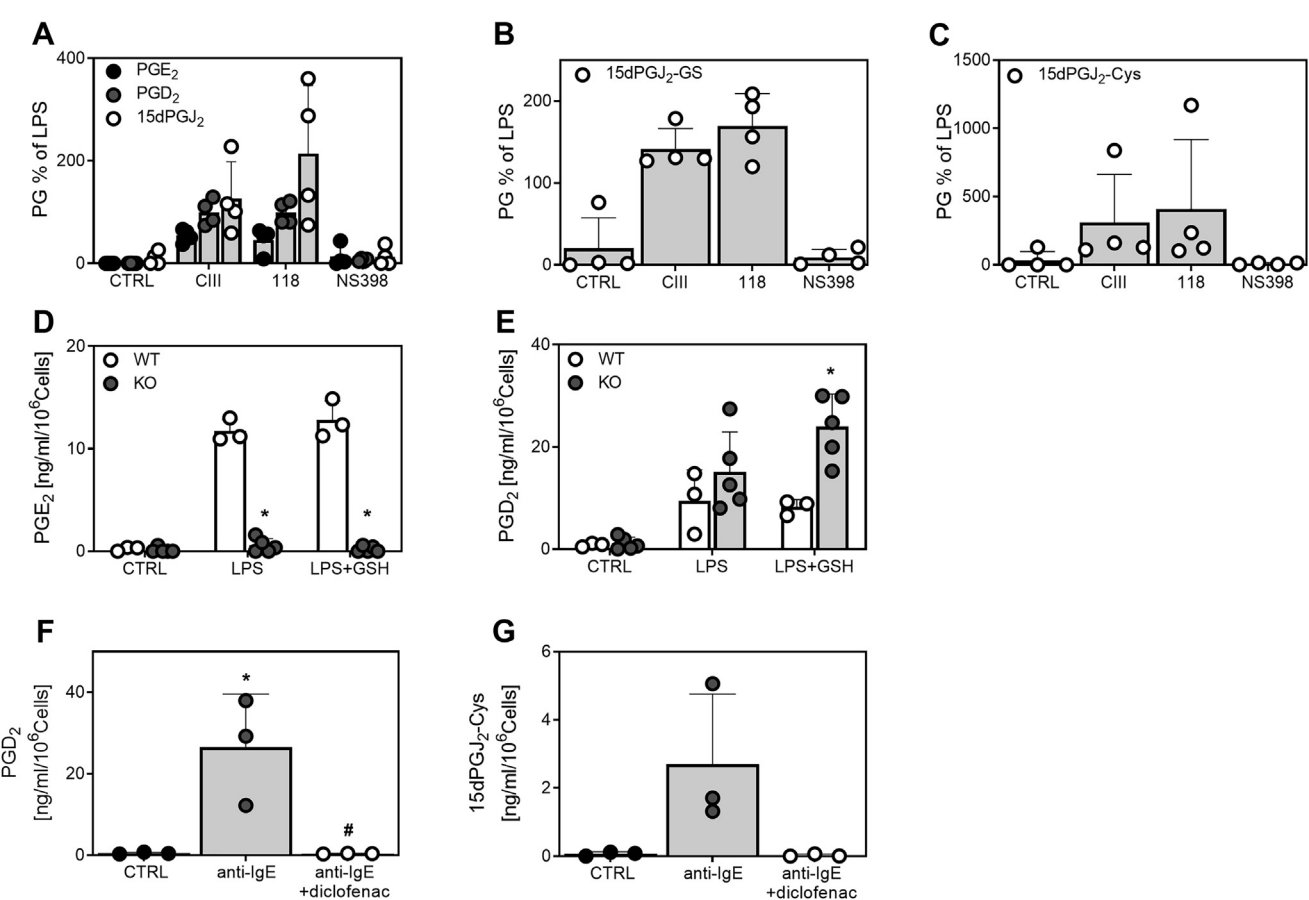
Effect of mPGES-1 depletion on the formation of PGE_2_, PGD_2_, and PGD_2_ metabolites in RAW264.7 cells, BMDM, and mast cells. (A–C) RAW264.7 cells were treated with LPS for 24 h in the presence or absence of mPGES-1 inhibitors CIII (10 μM) or 118 (1 μM). For comparison, the COX-2 inhibitor NS-398 (0.1 μM) was used. Prostaglandin (PG) levels are expressed as relative amounts to LPS stimulation in the absence of inhibitors. CTRL represents unstimulated cells. Data show four independent experiments. Comparison of PGE_2_ (D) and PGD_2_ (E) formation in BMDM from WT (n = 3) and mPGES-1 knock-out (KO, n = 5) mice treated with LPS or LPS and GSH for 24 h. CTRL represents unstimulated cells. ∗*P* < 0.05 indicates significant difference to WT. (F, G) CBMCs were stimulated with anti-IgE for 24 h in combination with the COX-1/COX-2 inhibitor diclofenac (1 μM) (n = 3). CTRL represents unstimulated cells. ∗*P* < 0.05 indicates significant difference to CTRL, ^#^*P* < 0.05 indicates significant difference to anti-IgE. COX, cyclooxygenase; CBMCs, cord-blood derived mast cells; BMDM, bone marrow derived macrophages; PGD_2,_ prostaglandin D_2_; LPS, lipopolysaccharides; 15dPGJ_2_-Cys, 15dPGJ_2_-cysteine; mPGES-1, microsomal prostaglandin E synthase-1.

We investigated whether treatment with mPGES-1 inhibitors affected the PGD_2_ pathway. RAW264.7 cells were stimulated for 24 h with LPS in combination with the selective mPGES-1 inhibitors CIII and 118 or the COX-2 inhibitor NS-398. Treatment with mPGES-1 inhibitors resulted in a 40%–50% reduction of PGE_2_ levels, with compound 118 reaching similar efficacy at a 10-fold lower concentration than CIII, indicating greater efficacy of compound 118 than CIII. PGD_2_ levels were not affected by inhibition of mPGES-1. The levels of 15dPGJ_2_, 15dPGJ_2_-GS, and 15dPGJ_2_-Cys tended to increase upon treatment with both mPGES-1 inhibitors, although the difference was not statistically significant. In contrast, COX-2 inhibition abolished the production of PGE_2_ and the analyzed PGD_2_ metabolites (Fig. 5A–C).

In addition, we probed the shunting to the PGD_2_ pathway in primary macrophages derived from bone marrow, by comparing WT and mPGES-1 KO mice. Primary macrophages from mPGES-1 KO mice treated with LPS or LPS in combination with GSH showed significantly reduced PGE_2_ levels (Fig. 5D). This was accompanied by a significant increase in PGD_2_ levels in mPGES-1 KO macrophages after treatment with LPS in combination with GSH (Fig. 5E). No significant shunting to PGF_2α_ or TXB_2_ formation was observed in macrophages from mPGES-1 KO mice (supplemental Fig. S5B, C). The levels of 15dPGJ_2_ and 15dPGJ_2_-conjugates were below quantification limit in these experiments.

Finally, we tested how COX inhibition affects the formation of PGD_2_ and the 15dPGJ_2_-Cys conjugate in human mast cells. COX-1/COX-2 inhibition with diclofenac blocked the formation of both PGD_2_ and 15dPGJ_2_-Cys (Fig. 5F, G). Human mast cells did not produce PGE_2_ and consequently, the mPGES-1 inhibitor 118 did not affect PGD_2_ and 15dPGJ_2_-Cys formation (data not shown).

## Discussion

**Fig. 6:**
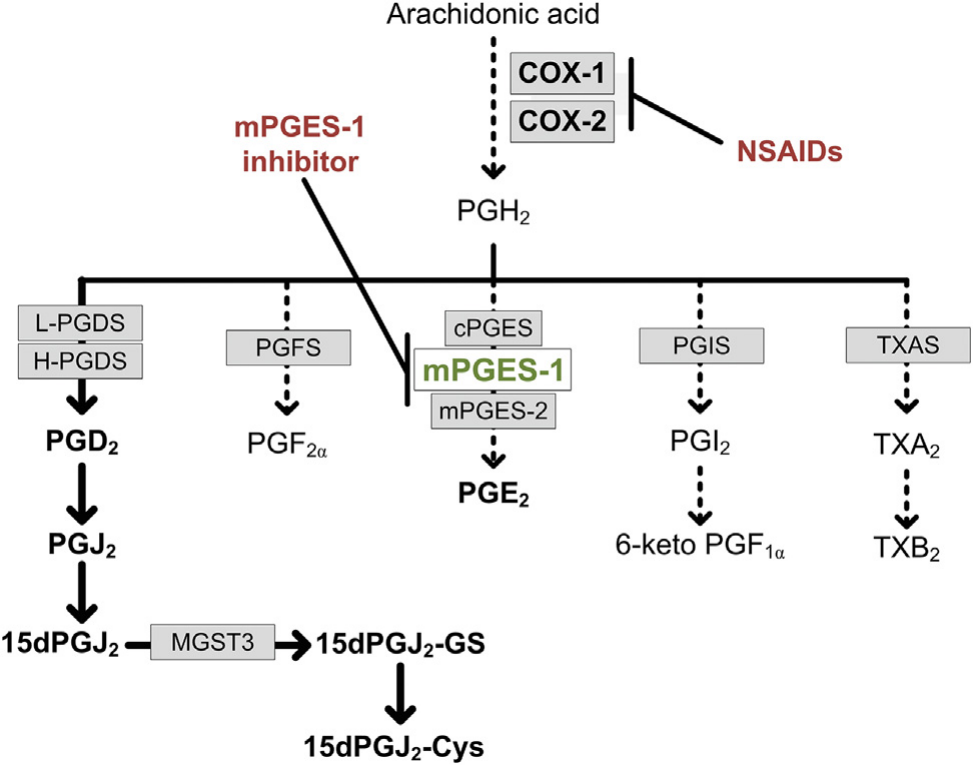
Schematic overview of prostanoid biosynthesis and changes in metabolism upon pharmacological inhibition of COX-1/COX-2 and mPGES-1. Arachidonic acid, released from membrane phospholipids by PLA_2_, is converted by cyclooxygenases (COX-1, COX-2) to PGH_2_. PGH_2_ serves as precursor for downstream synthases generating PGE_2_ (cytosolic PGE synthase, microsomal PGE synthase 1, microsomal PGE synthase 2), PGD_2_ (lipocalin PGD synthase, hematopoietic PGD synthase), PGF_2α_ (PGF synthase), PGI_2_ (PGI synthase), and TXB_2_ (TXA synthase). Inhibition of cyclooxygenases by NSAIDs targeting COX-1 and/or COX-2 blocks the formation of all downstream prostaglandins. Inhibition of mPGES-1, the inducible PGE_2_ synthase, blocks solely the formation of proinflammatory PGE_2_, while a redirection of PGH_2_ into PGD_2_, PGF_2α_, PGI_2_, or TXB_2_ may occur in a cell type and tissue-dependent manner. In macrophages, inhibition of mPGES-1 reinforces the PGD_2_ pathway including its downstream metabolite 15dPGJ_2_, which can be conjugated to GSH by MGST3 (microsomal glutathione S-transferase 3) generating 15dPGJ_2_-GS and 15dPGJ_2_-Cys. COX, cyclooxygenase; PGD_2,_ prostaglandin D_2_, PGE_2,_ prostaglandin E_2_; NSAID, nonsteroidal anti-inflammatory drug; mPGES-1, microsomal prostaglandin E synthase-1; 15dPGJ_2_-Cys, 15dPGJ_2_-cysteine; 15dPGJ_2_-GS, 15dPGJ_2_-glutathione.

mPGES-1 has shown promise as an alternative target for anti-inflammatory treatment strategies with improved selectivity and safety compared to traditional nonsteroidal anti-inflammatory drugs. The protective effect of mPGES-1 inhibition is thought to be due to the sole reduction of induced PGE_2_ and the associated upregulation of anti-inflammatory and cardio-protective prostanoids (38). In the present study, we demonstrate the biosynthesis of metabolites downstream of PGD_2_ in a murine macrophage cell line and in primary murine and human immune cells. 15dPGJ_2_ is an anti-inflammatory and proresolving lipid mediator with potent bioactivity, and our data contribute to a better understanding of its biosynthesis and metabolism under inflammatory conditions and upon inhibition of mPGES-1 (summary in Fig. 6).

We observed that 15dPGJ_2_ is conjugated to GSH and converted to a 15dPGJ_2_-Cys conjugate by RAW264.7 cells and mouse primary macrophages. GSH is synthesized in the cytosol, and more than 98% of GSH is in the thiol-reduced form, which allows intracellular nucleophile-electrophile interaction (39). In order to be conjugated to GSH and further metabolized, 15dPGJ_2_ must be exposed to cells, as demonstrated in our experiments by incubation of 15dPGJ_2_ in cell-free culture medium, which did not result in equivalent formation of 15dPGJ_2_-GS or 15dPGJ_2_-Cys, the latter even when GSH was added. The conversion of PGD_2_ to PGJ_2_ and 15dPGJ_2_ was shown to be independent of albumin (15). Similarly, we observed that the formation of 15dPGJ_2_ conjugates in RAW264.7 cells occurred independently of albumin (data not shown). Furthermore, inhibition of cellular transcription and translation blocked the formation of 15dPGJ_2_-GS in RAW264.7 cells. Taken together, these observations propose an enzyme-dependent conversion of 15dPGJ_2_ to the 15dPGJ_2_-GS and the 15dPGJ_2_-Cys metabolites. However, it is not clear which steps of 15dPGJ_2_ metabolism (GSH conjugation, reduction of the cyclopentenone ring, or removal of glutamic acid and glycine) are enzymatically regulated and which enzymes are involved. In addition, we cannot exclude the possibility that 15dPGJ_2_-Cys may be formed by direct reaction of 15dPGJ_2_ with cysteine. Several GSTs were suggested to be responsible for the formation of cyclopentenone-GS conjugates and in analogy to the leukotriene pathway, γ-glutamyl transpeptidases and dipeptidases might be catalyzing the generation of the 15dPGJ_2_-Cys metabolite (26, 40, 41). We found significantly enhanced formation of 15dPGJ_2_-GS catalyzed by the MGST3, an enzyme belonging to the MAPEG family (42). MGST3 shows both glutathione transferase and glutathione peroxidase activities (43), and RAW264.7 cells that lacked MGST3 were used to investigate if MGST3 is involved in the conjugation of 15dPGJ_2_ with GSH in intact cells. Knock out of MGST3 significantly reduced the formation of 15dPGJ_2_-GS and 15dPGJ_2_-Cys, demonstrating the catalytic activity of MGST3 in this pathway. We have focused here mainly on members of the MAPEG family, but there may be other GST, cytosolic and mitochondrial GST, involved in the metabolism of 15dPGJ_2_ with GSH (44). MGST3 expression has been described in human and mouse macrophages (44, 45) and in various tissues (e.g., human heart, brain, liver, kidney, pancreas, thyroid, testis, and ovary) (43). Because detection of MGST3 protein by Western blot is difficult, we assessed mRNA levels of MGST3, LTC4S, and MGST2 in RAW264.7 and human mast cells. We found MGST3 mRNA expression in both RAW264.7 cells and human mast cells. Treatment with an mPGES-1 inhibitor or a COX-inhibitor did not affect the expression levels of *Mgst3*, *Ltc4s*, and *Mgst2*, in stimulated RAW264.7 cells, with an overall reduction observed upon LPS stimulation. LPS has previously been shown to reduce LTC4S and MGST3 mRNA (46, 47). Others also showed that MGST3 protein levels were not affected by Kdo_2_-lipid A treatment in RAW264.7 cells, but tended to decline over time (45). In CBMCs, anti-IgE and treatment with diclofenac had no effect on the expression levels of *MGST2* and *MGST3*, which may indicate baseline expression of the enzymes as previously described (48). Even though mRNA expression of MGST3 decreased with LPS treatment, these results together with our observation that 15dPGJ_2_ can induce MGST3 mRNA and that the formation of the 15dPGJ_2_-conjugates requires de-novo protein biosynthesis suggest the involvement of MGST3 in the metabolism of 15dPGJ_2_ in immune cells.

Consistent with previous studies, we detected PGD_2_ as the predominant endogenous prostaglandin produced by RAW264.7 cells, with levels at 24 h twice that of PGE_2_. At the earlier time points (6–12 h), we found a comparably lower PGD_2_/PGE_2_ ratio, which may be explained by different stimuli and stimulus concentrations (49). We found the formation of 15dPGJ_2_, 15dPGJ_2_-GS, and 15dPGJ_2_-Cys in LPS-stimulated RAW264.7 cells following a kinetic profile consistent with the conversion of PGD_2_ to 15dPGJ_2_ and further to the GS-metabolites. However, we found lower levels of both metabolites in extracted cell pellets than in the supernatants. This could be explained by the rapid secretion of prostaglandins by RAW264.7 cells (50).

To further investigate the PGD_2_/15dPGJ_2_ pathway and identify 15dPGJ_2_ metabolites, we studied their production in primary mouse and human immune cells. In primary mouse macrophages, we found that PGD_2_ levels were higher than PGE_2_ levels 12 h after LPS stimulation, which converted to higher PGE_2_ levels after 24 h, similar to what was previously reported (49). We identified endogenous 15dPGJ_2_-Cys in primary murine macrophages upon LPS stimulation after 32 h. In addition, we found endogenously produced PGD_2_ and 15dPGJ_2_-Cys in human anti-IgE-stimulated mast cells. The 15dPGJ_2_-Cys conjugate was detected after 24 h of mast cell stimulation, likely due to superior stability or delayed formation of this metabolite as we did only detect traces of 15dPGJ_2_ and the 15dPGJ_2_-GS conjugate at this time point.

The formation of GSH conjugates with cyclopentenone prostaglandins and metabolites (i.e., PGA_2_, 9-deoxy-PGD_2_, PGJ_2,_ and 15dPGJ_2_) have been described previously in various cells (26, 51, 52, 53). HepG2 cells have been shown to produce the 15dPGJ_2_-Cys as final metabolite when incubated with exogenous 15dPGJ_2_ (28). However, with the exception of two studies in which the 15dPGJ_2_-GS was detected in vehicle-treated MCF7 cells and 15dPGJ_2_-like metabolites were detected in rat liver, respectively, there are no reports on the identification of endogenously formed 15dPGJ_2_-GS and 15dPGJ_2_-Cys metabolites (25, 53). Here, we described endogenous 15dPGJ_2_-GS and 15dPGJ_2_-Cys metabolites in murine macrophages and human mast cells and characterized the *in vitro* kinetics of 15dPGJ_2_ metabolism under inflammatory conditions. Although GSH is the most abundant thiol in the cell, we cannot exclude retro-Michael addition reactions as well as conjugation of 15dPGJ_2_ to other thiol-containing proteins. Given that no internal standard normalization was applied and quantification of the 15dPGJ_2_-Cys conjugate was based on an in-house prepared standard, the levels we report should be considered as semiquantitative. Thus, in line with results by others (9, 52, 54), our data support the endogenous formation of 15dPGJ_2_ and GSH-conjugation under inflammatory conditions. Whether the metabolism of 15dPGJ_2_ into the 15dPGJ_2_-GS and 15dPGJ_2_-Cys conjugate is mainly a detoxification process or results in bioactive compounds that potentially activate nuclear receptors such as PPARγ and retinoid X receptor and how this metabolism might be controlled and related to pathogenesis remains to be further elucidated.

Depending on the inflammatory milieu, PGD_2_ and its metabolites are considered as proresolving lipids (55, 56, 57), and suppression of these lipids by COX inhibitors might interfere with successful resolution, contributing to chronic inflammation. Recent studies highlight a possible impact of mPGES-1 inhibition and PGD_2_ metabolites on the polarization of macrophages toward an anti-inflammatory phenotype (9, 10, 12). Here we show that, in contrast to COX-2 inhibition, inhibition of mPGES-1 did not reduce PGD_2_ metabolite formation. Instead, after treatment with mPGES-1 inhibitors, we observed a tendency for increased production of these lipids in RAW264.7 cells and significant shunting to PGD_2_ in primary macrophages from mPGES-1 KO mice stimulated with LPS and GSH. Further, in human mast cells, we found COX-derived PGD_2_ and the metabolite 15dPGJ_2_-Cys, while no PGE_2_ was detected. These results are consistent with previous publications showing that expression of mPGES-1 in human mast cells is not induced by cytokines or IgE stimulation (58, 59).

## Conclusion

In conclusion, our data show the formation of 15dPGJ_2_-conjugates in activated mouse macrophages and human mast cells, and we identify MGST3 as essential for 15dPGJ_2_-conjugates formation. Moreover, inhibition of mPGES-1 maintained PGD_2_ metabolites, in contrast to inhibition of COX, which abolished their formation. Overall, the results presented provide new insights into the biosynthesis of lipid metabolites derived from the PGD_2_/15dPGJ_2_ pathway and motivate further research on their role in inflammation.

## Data Availability

The data supporting the findings of this study are contained within the manuscript and the supplementary information file.

## Supplemental Data

This article contains supplemental data.

## Conflict of Interest

The authors declare that they have no known competing financial interests or personal relationships that could have appeared to influence the work reported in this paper. P.-J. J. is engaged in Gesynta Pharma AB, a company that develops mPGES-1 inhibitors.
