# Supplementary material for: Biosynthesis of prostaglandin 15dPGJ2 -glutathione and 15dPGJ2-cysteine conjugates in macrophages and mast cells via MGST3
Source: J Lipid Res. 2022 Nov 9;63(12):100310. doi: 10.1016/j.jlr.2022.100310 (PMC9792570; doi:10.1016/j.jlr.2022.100310)
Supplement: Supplementary [file mmc1.docx]

**Biosynthesis of prostaglandin 15dPGJ_2_ -glutathione and -cysteine conjugates in macrophages and mast cells via MGST3**

Julia Steinmetz-Späh^1^, Jianyang Liu^1^, Rajkumar Singh^3^, Maria Ekoff^2^, Sanjaykumar Boddul^1^, Xiao Tang^3^, Filip Bergqvist^1^, Helena Idborg^1^, Pascal Heitel^4^, Elin Rönnberg^2^, Daniel Merk^4^, Fredrik Wermeling^1^, Jesper Z. Haeggström^3^, Gunnar Nilsson^2^, Dieter Steinhilber^4^, Karin Larsson^1^, Marina Korotkova^1^, Per-Johan Jakobsson^1^.

^1^Division of Rheumatology, Department of Medicine, Solna, Karolinska Institutet and Karolinska University Hospital, SE-171 76 Stockholm, Sweden

^2^Immunology and Allergy division, Department of Medicine, Solna, Karolinska Institutet and Karolinska University Hospital, SE-171 76 Stockholm, Sweden

^3^Division of Physiological Chemistry 2, Department of Medical Biochemistry and Biophysics, Biomedicum 9A, Karolinska Institutet SE-171 65 Stockholm, Sweden

^4^Institute of Pharmaceutical Chemistry, Goethe-University Frankfurt, Max-von-Laue-Straße 9, 60438, Frankfurt, Germany

**Table S1: Multiple reaction monitoring method parameters for analysis of 15dPGJ_2_-conjugates by LC-MS/MS.**

| **Compound** | **Ionization mode** | **Precursor ion (*m/z*)** | **Product ion (*m/z*)** | **Cone voltage (V)** | **Collision energy (V)** | **RT (min)** |
| --- | --- | --- | --- | --- | --- | --- |
| 15dPGJ_2_-GS | ES+ | 624.4 | 179.2 | 40 | 38 | 7.25 |
| 15dPGJ_2_-GS | ES+ | 624.4 | 308.3 | 40 | 26 | 7.25 |
| 15dPGJ_2_-GS | ES+ | 624.4 | 317.4 | 40 | 28 | 7.25 |
| 15dPGJ_2_-Cys | ES+ | 440.4 | 301.2 | 15 | 12 | 7.14 |
| 15dPGJ_2_-Cys | ES+ | 422.4 | 301.2 | 15 | 12 | 7.14 |


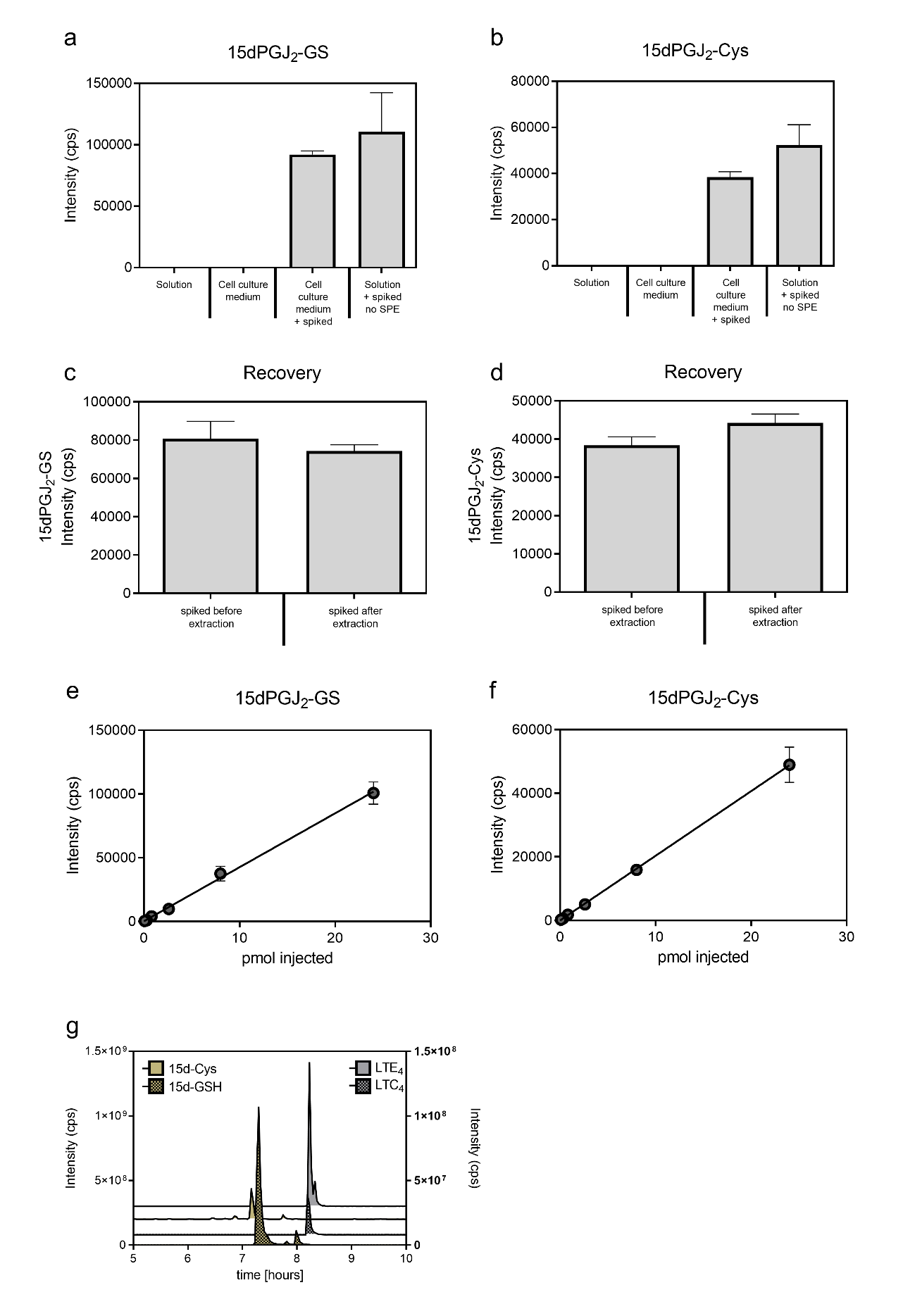


**Figure S1**: **Analysis of** **15dPGJ_2_ metabolites, LTC_4_ and LTE_4_ by LC-MS/MS**. (a, b) The matrix effect was determined by comparing spiked conjugates in cell culture medium or 20 % acetonitrile referred to as solution (n=2-3). The observed signal suppression was 17 % and 26 % respectively. (c, d) The recovery rate was determined by spiking 15dPGJ_2_-GS or 15dPGJ_2_-Cys into cell culture medium before or after solid-phase extraction (n=3). Full recovery was observed for 15dPGJ_2_-GS and 87 % recovery for 15dPGJ_2_-Cys. (e, f) We found good linearity of the LC-MS/MS method for both analytes (15dPGJ_2_-GS, R^2^=0.998 and 15dPGJ_2_-Cys, R^2^=0.999) in solution over a dynamic range of 0-24 pmol injected and the lower limit of quantification to be 0.1 pmol injected (SN>10) (n=2). (g) Total ion chromatograms of 15dPGJ_2_-GS (*m/z* 624.4, ES+), 15dPGJ_2_-Cys (*m/z* 422.4, ES+), LTC_4_ (*m/z* 624.8, ES-) and LTE_4_ (*m/z* 438.3, ES-) were analyzed. The commercially obtained or in-house generated compounds were reconstituted or diluted in 20% acetonitrile and injected on a 50 × 2.1 mm Acquity UPLC BEH C18, 1.7 μm column (Waters) at a flowrate of 0.6 mL/min. The compounds were separated in a 13 min analytical gradient with 0.05% FA/MQ water as mobile phase A and 0.05% FA/acetonitrile as mobile phase B. The intensity corresponds to counts per second (cps) measured. The elution profiles reveal separation (1 minute) of the 15dPGJ_2_-metabolites and leukotrienes, as well as differentiation of the compounds by mass for LTE_4_.


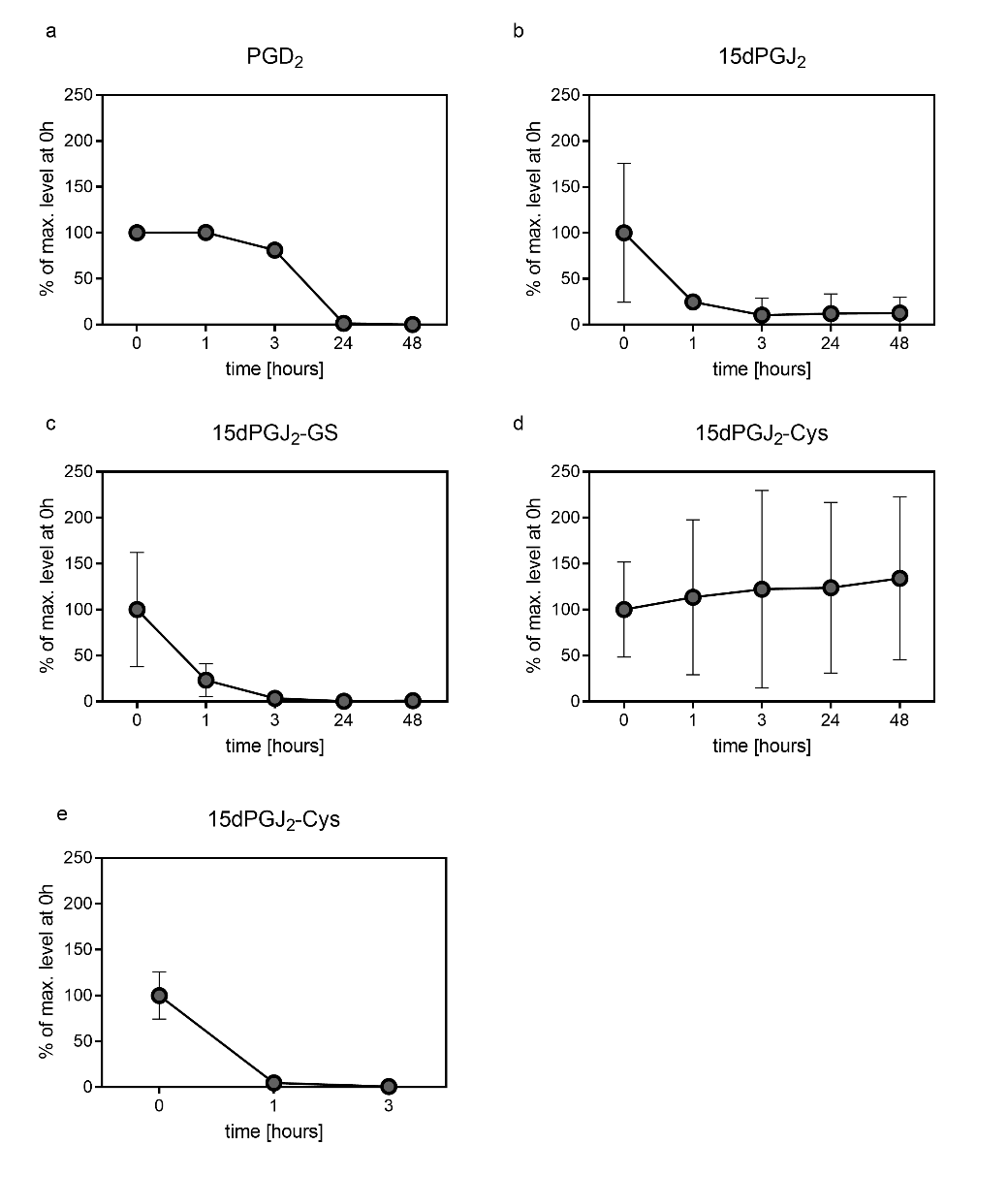


**Figure S2: Stability of PGD_2_, 15dPGJ_2_, 15dPGJ_2_-GS and 15dPGJ_2_-Cys in plasma and pharmacokinetic of 15dPGJ_2_-Cys in mice**. (a, b, c, d) Compounds were spiked into human plasma and remaining levels were measured after indicated time points. The data are expressed as percentage of maximum plasma levels at the 0 h time point. Data show mean (4-5 technical replicates) of 1 donor for PGD_2_ and mean ± SD of 3 donors for 15dPGJ_2_, 15dPGJ_2_-GS and 15dPGJ_2_-Cys. (e) Plasma levels of 15dPGJ_2_-Cys after s.c injection of 15dPGJ_2_-Cys (5µg) in mice.


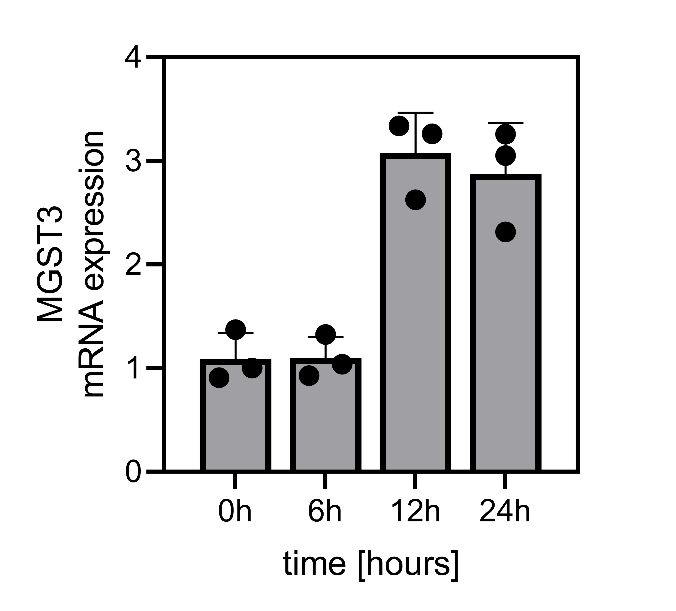


**Figure S3: MGST3 mRNA expression upon 15dPGJ_2_ treatment of RAW264.7 cells**. Relative expression MGST3 was measured by qRT-PCR in RAW264.7 cells treated with 15dPGJ_2_ for indicated time-points. β-actin was used as an internal control to normalize target gene expression. Data are presented as mean ± SD of three independent experiments.


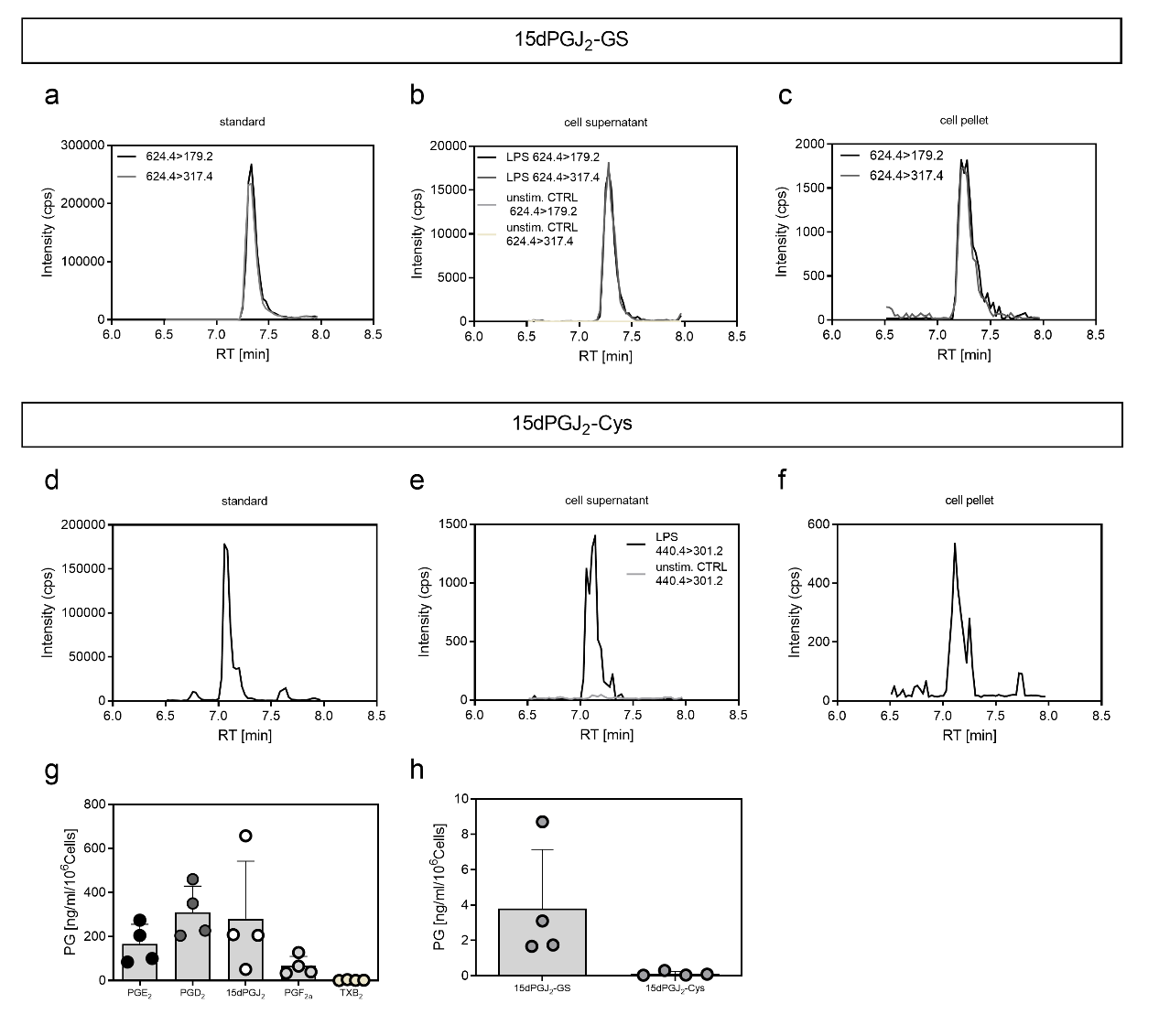


**Figure S4**: **Endogenous formation of prostaglandins, 15dPGJ_2_ and its metabolites by LPS stimulated RAW264.7 cells after 24 h.** (a, d) Representative MRM chromatograms for 15dPGJ_2_-GS (upper panel) and 15dPGJ_2_-Cys (middle panel) synthesized standards in 20% acetonitrile. (b, e) Endogenously detected conjugates in cell supernatants of LPS (5 µg/mL) stimulated or unstimulated cells and (c, f) corresponding cell pellets of LPS stimulated cells. (g, h) Quantification of prostanoids (PG) in supernatants of LPS (2 µg/mL) stimulated RAW264.7 cells after 24 h. Data are expressed as mean ± SD of four independent experiments.


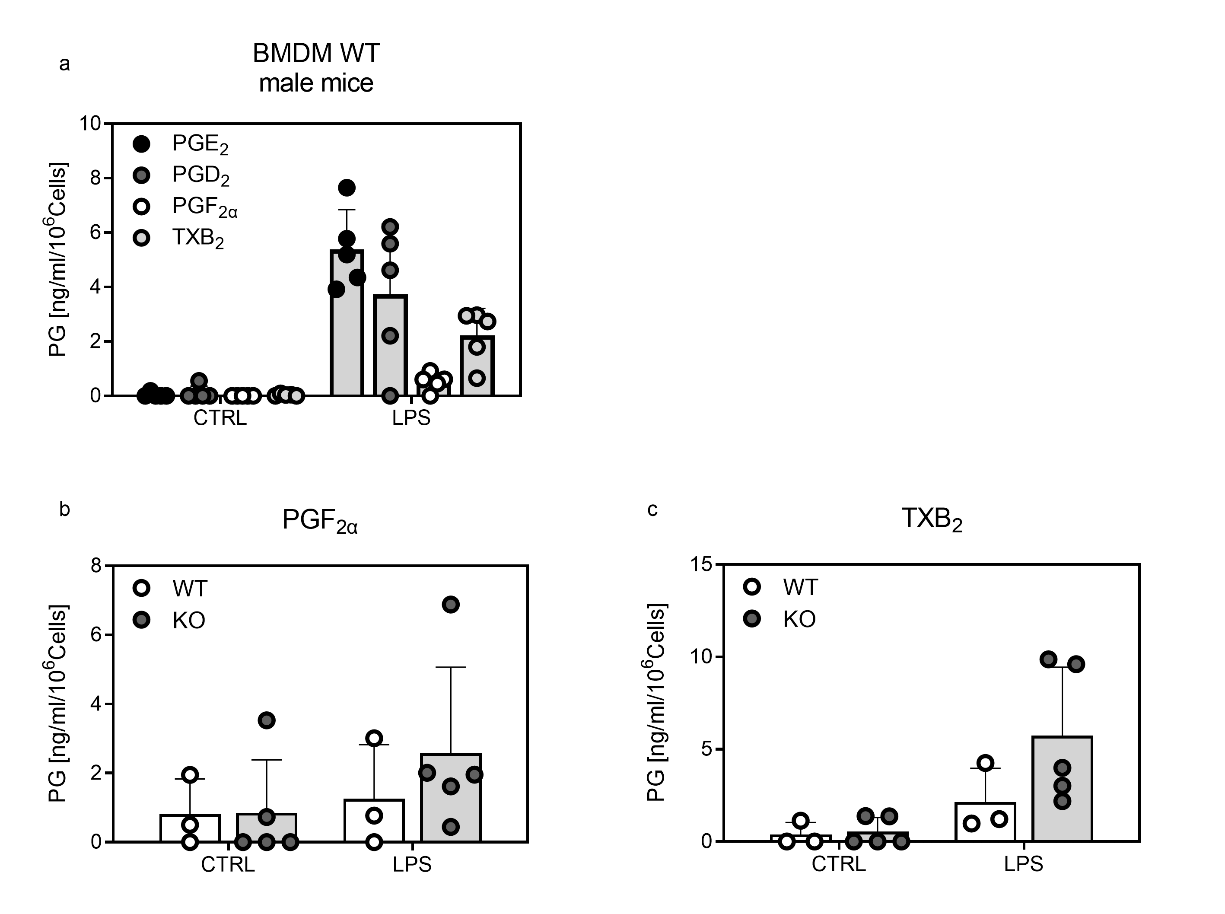


**Figure S5:** **Analysis of PGE_2_, PGD_2_, PGF_2α_, and TXB_2_ in murine primary cells**. BMDM from wild type (WT) and mPGES-1 knock-out mice were treated with 2 µg/mL LPS or fresh culture medium (CTRL) for 24 h. (a) Supernatants were extracted, and prostanoids were quantified comparing unstimulated and stimulated macrophages from male WT (n=5) mice. PGF_2a_ (b) and TXB_2_ (c) were quantified comparing levels in macrophages from female WT (n=3) and mPGES-1 KO (n=5) mice. Data are expressed as mean ± SD.

**Figure S6**: **Identification of 15dPGJ_2_ and 15dPGJ_2_-GS metabolites in human primary cells**. CBMCs (cord-blood derived mast cells, 1.0 M / ml) were stimulated with anti-IgE for 24 h. Supernatants were extracted and 15dPGJ_2_, 15dPGJ_2_-GS and 15dPGJ_2_-Cys were analyzed. Chromatogram of the 15dPGJ_2_ standard (a), endogenous 15dPGJ_2_ in unstimulated (d) and anti-IgE stimulated cells (g) is shown in the first column. The second and third column show the chromatograms of 15dPGJ_2_-GS (*m/z* 624.4>308.3) and 15dPGJ_2_-Cys (*m/z* 422.4>301.2) standards (b and c respectively) and endogenous compounds in unstimulated (e and f respectively) and anti-IgE stimulated (h and i respectively) CBMCs. Data shows chromatograms of one representative donor.


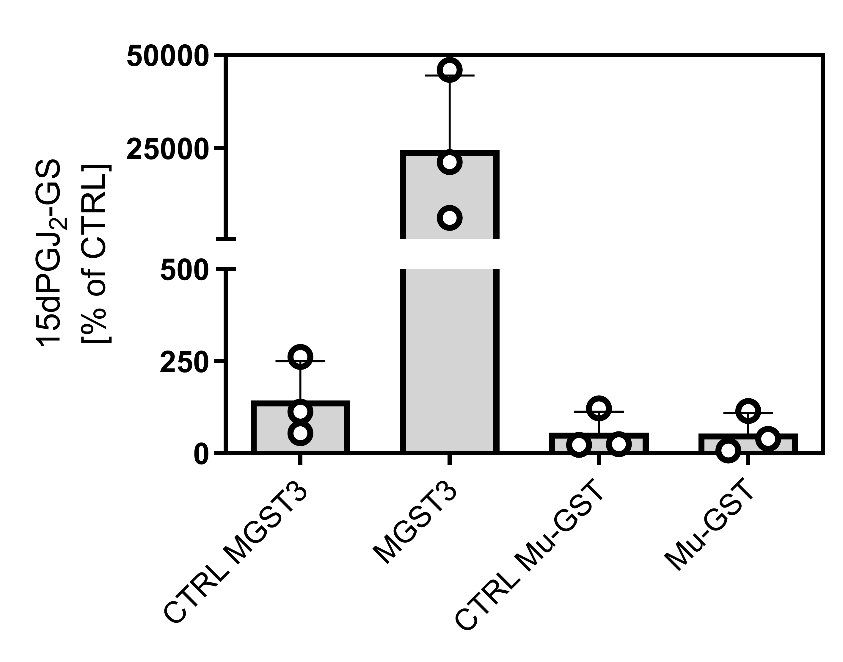


**Figure S7: Formation of 15dPGJ_2_-GS in the presence of Mu-GST and MGST3.** In parallel control reactions without enzyme were performed for both enzymes tested (CTRL). Data shows one experiment performed in triplicates (mean ± SD). Analyte was quantified with LC-MS/MS (*m/z* 624.4>317.4).
